# Structural Analysis of Tin-Substituted High-Entropy Li-Garnet Electrolytes for Solid-State Batteries

**DOI:** 10.1021/acsorginorgau.5c00021

**Published:** 2025-04-30

**Authors:** Benjamin Zimmermann, Till Fuchs, Johannes Westphal, Jürgen Janek, Maren Lepple

**Affiliations:** † Institute of Inorganic and Analytical Chemistry, 54250 Justus Liebig University Giessen, Heinrich-Buff-Ring 17, Giessen 35392, Germany; ‡ Institute of Physical Chemistry, Justus Liebig University Giessen, Giessen 35392, Germany; § Center for Materials Research, Justus Liebig University Giessen, Giessen 35392, Germany

**Keywords:** high-entropy, lithium garnet, Li_7_La_3_Zr_2_O_12_, solid electrolyte, synthesis, characterization, tin

## Abstract

Lithium garnets offer promising structural and electrochemical properties and could be used in all solid-state lithium batteries replacing liquid electrolytes. They can operate in a wide electrochemical voltage window and show high ionic conductivities (>10^–4^ S cm^–1^). The best-studied lithium garnet is Li_7_La_3_Zr_2_O_12_ (LLZO), which is known to undergo a transition from an ordered, tetragonal form to a disordered cubic modification at elevated temperatures. This is crucial, as the cubic modification offers about 2 orders of magnitude higher ionic conductivities. Applying the high-entropy concept to this material facilitates the stabilization of the cubic structure at ambient conditions. In this work, four different lithium garnet compositions based on Li_6_La_3_Zr_0.5_Nb_0.5_Ta_0.5_Hf_0.5_O_12_ have been synthesized by mixing Zr^4+^, Nb^5+^, Ta^5+^, and Hf^4+^ by Sn^4+^, respectively, using two different solid-state approaches. They have been characterized by X-ray diffraction, energy-dispersive X-ray spectroscopy, and impedance spectroscopy to analyze the influence of synthesis parameters and composition on phase purity, elemental distribution, and ionic conductivity. It was found that combining calcination and sintering into one process yields a higher density and ionic conductivity than splitting it into two with intermediate regrinding of the material. Impedance data indicate an increase in ionic conductivity when substituting pentavalent ions for tetravalent ones due to the resulting higher concentration of mobile charge carriers in the structure, compared to Li_6_La_3_Zr_0.5_Nb_0.5_Ta_0.5_Hf_0.5_O_12_.

## Graphical abstract

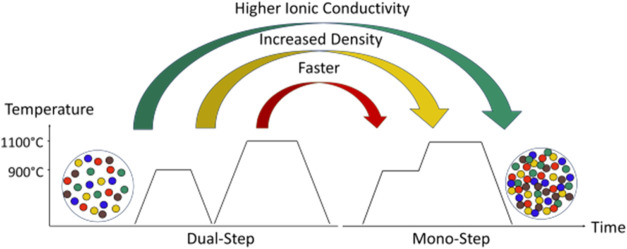

## 1 Introduction

Since the introduction of the modern Li-ion battery by Sony in 1991, liquid electrolytes based on ethylene carbonate and LiPF_6_ have been the standard commercial solution.^1,2^ These liquid electrolytes have the disadvantage of high flammability and instability at high voltages. Over time, there is a loss of active lithium and maximum capacity caused by the formation of a SEI (solid electrolyte interphase) and cathode degradation. Also, after decades of research, the maximum potential of this setup has been reached with only minimal improvements possible as lithium metal anodes cannot be used due to dendrite risk.^3–5^ To overcome these problems, solid electrolytes have been receiving increasingly more attention in recent years. A promising candidate is the Li_7_La_3_Zr_2_O_12_ (LLZO) lithium garnet. It combines good thermal stability, a wide electrochemical window,^6–8^ compatibility with a Li metal anode,^9^ and competitive ionic conductivities (10^–4^–10^–3^ S cm^–1^). The properties of LLZO strongly depend on the crystal structure, composition, and synthesis conditions. LLZO exists in an ordered tetragonal phase (*I*4_1_/*acd*) with three distinct Li positions (8a, 16f, 32g) and a disordered cubic high-temperature phase (*Ia*-3*d*), where the Li^+^ ions occupy the 24d and 96h sites.^10^ The disordered and thus mobile Li^+^ ions enable the cubic structure to exhibit ionic conductivities around 2 orders of magnitude higher than the tetragonal structure. There is a strong push in research to stabilize the cubic structure at low temperatures, either by substituting elements or by refining the synthesis parameters. Wet chemical approaches like sol–gel synthesis or co-precipitation have the advantage of much lower synthesis temperatures, which lead to less lithium loss. This way, the stoichiometry can be controlled much more precisely.^11^ However, they require additional chemicals (e.g., solvents, acids, and bases) and are more time-consuming because the reactants require additional drying steps. The solid-state synthesis offers fewer processing steps and a better atom economy.

The garnet structure offers many possibilities for compositional variability.^12^ This is due to having three distinct cation sublattices inside of the crystal structure. The general formula of lithium garnets can be described as A*_*x*_* B_3_C_2_O_12_ (*x* = 5–7), where A, B, and C are coordinated by eight, four, or six oxygen atoms, respectively. Suitable elements for each position in LLZO were determined by Miara et al.^13^ via density functional theory (DFT) calculations. The main element for the A position is lithium, but elements like Al and Ga can also be used to manipulate the Li content, as every Al^3+^ or Ga^3+^ cation replaces three Li^+^ ions and thereby introduces two vacancies. However, especially aluminum, is blocking the Li^+^ conduction paths and can thus reduce the ionic conductivity.^14,15^ On the other hand, unintentional incorporation of a small amount of Al^3+^ in earlier works using alumina crucibles for heat treatments is responsible for the stabilization of the cubic phase.^16^ The B position can be occupied by La or most of the lanthanides. The C position can be occupied by a variety of tetravalent and pentavalent ions like Zr^4+^, Nb^5+^, Ta^5+^, Hf^4+^, Sn^4+^, Ti^4+^, or Ce^4+^.^17^ Most commonly, however, the octahedral site, which the tetravalent zirconium cation occupies, is manipulated. Literature noted examples are Li_7–2*x*_La_3_Zr_2‑*x*_Mo_*x*_O_12_,^11^ Li_7_La_3_Zr_2‑*x*_Ti_*x*_O_12_,^18^ Li_7.1_La_3_Zr_1.9_Cr_0.1_O_12_,^19^ and Li_7_La_3_Sn_2_O_12_.^20^ The reason for the high interest is the position’s prime influence on the tetragonal to cubic phase transition and thus the ionic conductivity.^6,21^ This is explained by the structure of Li-garnets, which can be described by corner-sharing octahedra. Changing the octahedra forming ions thus has a big impact on the framework of the crystal structure and the observed transformations. It is also possible to create Li vacancies by using supervalent ions on the B or C sites without obstructing the migration paths, which could happen by directly substituting Li with Al.^15^

Fu and Ferguson^22^ showed that it is possible to go beyond doping levels of additional elements and synthesized a garnet with four cations on the C site: Li_6_La_3_Zr_0.5_Nb_0.5_Ta_0.5_Hf_0.5_O_12_. This approach shows that adding multiple elements in equimolar concentrations is a viable strategy for stabilizing the cubic polymorph of Li-garnets and enables the concept of *high entropy* for these materials. The high-entropy concept was originally established in metal alloys as a means to improve desirable properties^23^ and was then transferred to ceramic systems.^24,25^ Through introduction of multiple cations or anions on the same sublattice of a crystal structure, disorder is introduced into the system. To achieve the maximum configurational entropy, elements must be distributed statistically and in equimolar ratios in the lattice. The entropy of mixing can be calculated as follows:

$$\Delta S_{\mathrm{conf}}=-R\sum_{i=1}^{N}c_{i}\cdot \ln(c_{i})$$

With *R* being the ideal gas constant and *c*_*i*_ being the fractional composition of component *i*. Since many factors can influence this distribution, like ion radius, thermodynamic factors, and preferential phase formation (e.g., BCC vs FCC), it is important to prescreen possible element combinations. One of the methods for this prescreening is the estimation of the so-called “atomic size factor” δ

1 $$\delta =\sqrt{\sum_{i=1}^{N}c_{i}{\left(\frac{r_{i}}{\sum_{i=1}^{N}\;c_{i}\cdot r_{i}}\right)}^{2}}$$

with *c_i_* = concentration of element *i* (regarding a specific sublattice) and *r_i_* = its respective radius. δ decreases the closer the ionic radii of the ions on the same sublattice are. This increases the probability of successful substitution. The atomic size factor is also a concept first applied to high-entropy alloys (HEAs)^23^ but has since been used to predict the stability of multicomponent oxide systems. Multiple groups applied the concept on high-entropy rare earth zirconates with a fluorite or pyrochlore structure.^26,27^ They found a limit of δ to be between 4.5 and 5.29%. Above this value, the formation of dual-phase samples was observed. They also showed, however, that δ is not the only important descriptor regarding the ability to form single-phase compounds.

Applying these concepts to lithium garnets may benefit the stabilization of the cubic modification by further boosting its inherent disorder in the structure. As described above, the inherent disorder of the Li^+^ ions is key in enabling sufficient ion mobility and thus further benefits from incorporating additional disorder through the high-entropy approach. This, in turn, could enhance the electrochemical properties of the material. The incorporation of multiple elements also allows easier tailoring of properties by changing redox potentials, charge variance or changing the framework due to changes in ionic radii. This multitude of effects is called the *cocktail effect*.^28^ Challenges comprise the transferability of parameters like δ from pyrochlore structures to garnets since no widespread studies have been done so far on garnet structures. The compositional space is also limited by charge compensation and ionic radius match to ensure the formation of the desired structure.

In this work, tin has been chosen as the main element to substitute the cations on the C site of the already published high-entropy garnet Li_6_La_3_Zr_0.5_Nb_0.5_Ta_0.5_Hf_0.5_O_12_.^22^ The ionic radius of Sn^4+^ is very similar to those of the other cations sharing the same sublattice. This minimizes the value for δ and should increase the probability of the formation of single-phase samples. Chen et al.^29^ reported that elements with high valency are beneficial to Li^+^ ion conduction, which Sn^4+^ provides, while also being able to balance charge inhomogeneities by being reduced to Sn^2+^. Earlier reports about tin-containing lithium garnets focused on increasing the Li content by substituting Zr but could only obtain the tetragonal phase as the cubic phase would either transform back to the tetragonal phase or a high fraction of Li_2_SnO_3_ impurity would form.^20^ More recent reports attempted the stabilization of the cubic phase by intentionally incorporating small amounts of Al^3+^.^30^

Here, four different cubic tin-modified Li-garnets have been synthesized by solid-state reaction as phase-pure materials without the use of aluminum to stabilize the cubic structure. According to the formula for the configurational entropy provided above, the calculated values for the synthesized compounds Li_6_La_3_ **Sn**_0.5_Nb_0.5_Ta_0.5_Hf_0.5_O_12_ and Li_6_La_3_Zr_0.5_Nb_0.5_Ta_0.5_ **Sn**_0.5_O_12_ as well as Li_6.5_La_3_Zr_0.5_ **Sn**_0.5_Ta_0.5_Hf_0.5_O_12_ and Li_6.5_La_3_Zr_0.5_Nb_0.5_ **Sn**_0.5_Hf_0.5_O_12_ are 2.66R and 2.73R, respectively, whereas the approximate Li occupancies on the different sublattices were taken from literature.^31,32^ Thus, the compounds fulfill the minimum threshold of 1.5R to be determined “high-entropy”. The calculated values for δ exceed the upper limit reported for pyrochlore systems. The influence of synthesis conditions on phase purity has been analyzed via X-ray diffraction (XRD) and scanning electron microscopy (SEM) equipped with energy-dispersive X-ray spectroscopy (EDS), showing the good solubility of the elements within the chosen composition range. Their ionic conductivity was characterized by electrochemical impedance spectroscopy (EIS).

## 2 Experimental Section

### 2.1 Synthesis

Lithium garnets with nominal compositions of Li_6_La_3_ **Sn**_0.5_Nb_0.5_Ta_0.5_Hf_0.5_O_12_ (DS1/MS1), Li_6.5_La_3_Zr_0.5_ **Sn**_0.5_Ta_0.5_Hf_0.5_O_12_ (DS2/MS2), Li_6.5_La_3_Zr_0.5_Nb_0.5_ **Sn**_0.5_Hf_0.5_O_12_ (DS3/MS3), and Li_6_La_3_Zr_0.5_Nb_0.5_Ta_0.5_ **Sn**_0.5_O_12_ (DS4/MS4) were synthesized. Powders of Li_2_CO_3_ (≥99%, Roth), La_2_O_3_ (≥99.99%, ChemPur), ZrO_2_ (≥99.9%, ChemPur), Nb_2_O_5_ (≥99.9%, ChemPur), Ta_2_O_5_ (≥99.9%, ChemPur), HfO_2_ (≥99.95%, ChemPur) and SnO_2_ (≥99%, ChemPur) were mixed in stoichiometric ratios, respectively. Ten wt % excess of Li_2_CO_3_ was used to counteract lithium loss at high temperatures. The powders were ground in an agate mortar for 15 min and pressed into 10 mm pellets using isostatic pressure of 4 tons for 10 min. The pellets were placed in magnesium oxide crucibles to prevent Al contamination at high temperatures. The synthesis was carried out in two ways: (i) The pellets were fired in a box furnace in the air at 900 °C for 8 h, then, after cooling down, the grinding and pressing step was repeated. The sintering was carried out at 1100 °C for 12 h (dual-step, DS). (ii) The second method was heating the pellets at 900 °C for 8 h and then at 1100 °C for 12 h without intermediate cooling and grinding (mono-step, MS). Heating and cooling rates were 150 K/h for all temperature changes. The synthesis parameters were chosen close to proven methods reported in the literature to ensure a better comparability between results.^22,33^

### 2.2 Sample Preparation

Scanning electron microscope measurements were conducted on fragments of pellets, which were obtained by breaking off a piece of the sintered pellet using a mortar and pestle. The fragments were arranged in such a way that the original outer surface area of the pellet could be studied. The chosen pieces are placed on a sticky carbon pad and sputtered with ∼5 nm of carbon to ensure good electrical conductivity.

In preparation for the electrochemical impedance spectroscopy, pellets were polished with SiC sandpaper in Ar atmosphere, then placed in a sample mask, and subsequently coated on both sides with a ∼120 nm thick layer of gold using a custom-made setup at a rate of 0.2 nm/s. After contacting the gold electrodes with nickel tabs, the stack was sealed in a pouch cell under a vacuum.

### 2.3 Characterization

Powders were structurally characterized via powder X-ray diffraction (PXRD) using a Panalytical X’Pert Pro and Cu Kα radiation at 40 kV/40 mA with Bragg–Brentano geometry. The measuring range was from 10 to 75° with a step size of 0.0015° and time per step of 60 s with a 1° slit and 10 mm beam mask. Density of the samples was measured with whole pellets on an Anton Paar Ultrapyc 5000 gas pycnometer using helium as a medium. The densities were compared to theoretical densities obtained by Rietveld refinement done using GSAS 2 software.^34^ Morphology and elemental composition were studied with a scanning electron microscope (SEM) GeminiSEM 560 from Zeiss with a X-MAX Extreme detector by Oxford Instruments. The accelerating voltage was 1/10 kV with a working distance of 3.5/8.5 mm for normal pictures and the elemental mapping, respectively.

Temperature dependent electrochemical impedance spectroscopy was carried out using a VMP 300 potentiostat by BioLogic controlled by EC Lab (V11.2) between −40 and 40 °C. The temperature was controlled using a WKL 64 climate chamber by WEISS with a 1 h equilibration step after temperature changes. EIS was carried out between 7 MHz and 1 Hz with an excitation voltage of 10 mV. For data fitting, RelaxIS 3 by RhD Instruments was used.

## 3 Results and Discussion

### 3.1 Structural Analysis and Morphology

In Figure 1a,b, the XRD patterns of all sintered samples are given. The sample DS2 synthesized with the dual-step route in Figure 1a clearly shows La_2_O_3_ as an impurity, most notably at around 2Θ = 30°. The La_2_O_3_ impurity most likely originates from lithium loss occurring at elevated temperatures. Due to the repeated heating process, the samples spent a longer period at these high temperatures, which may lead to an unfavorable shift in stoichiometry if not enough excess lithium is present. Figure 1b shows the XRD patterns for the synthesized tin-garnet samples using the mono-step route. The cubic garnet reference (PDF 98–018–2312) and sample are in good agreement with each other, and no other phases were observed. The angles between 2Θ = 50° and 60° only show the three peaks typical for the cubic garnet phase.^21^ A shift in peak positions is observed, which we explain by the additional elements compared to those of LLZO. This is depicted in Figure 1c. The peak shift to higher angles indicates a decrease in the lattice parameter. Rietveld refinement was done to calculate the change in lattice parameters in comparison to Li_7_La_3_Zr_2_O_12_ and the theoretical densities. The resulting graph for the MS1 sample is depicted in Figure 1d as an example. The calculated lattice parameters of all samples from the Rietveld refinement are listed in Table 1, and the resulting graphs are depicted in Figures S1 and S2 in the SI. When comparing these values with the literature data for cubic LLZO with *a* = 12.94384 Å,^16^ the lattice parameters for the tin garnets are indeed smaller. By taking into account the ionic radii of the cations in question: Zr^4+^ (0.72 Å), Nb^5+^ (0.64 Å), Ta^5+^ (0.64 Å), Hf^4+^ (0.71 Å), and Sn^4+^ (0.69 Å)^35^ the pattern follows the expected trend. Since zirconium is the biggest ion, reducing its fraction by adding other elements should lead to a general reduction in the lattice parameter, as observed in the four synthesized materials.

**Figure 1:**
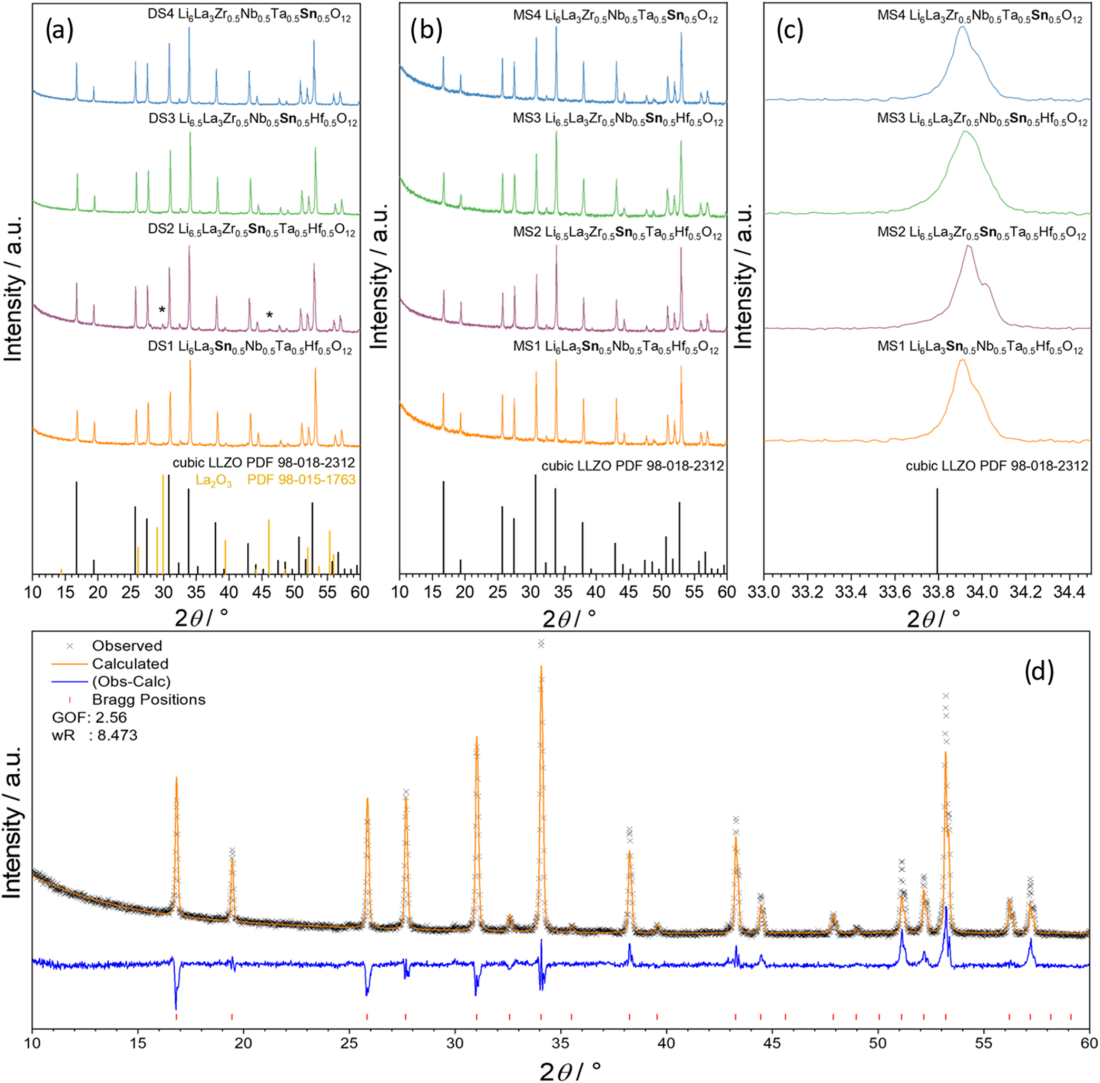
X-ray diffraction patterns of the samples obtained with dual-step (a) and mono-step sintering (b) with zoom (c) and Rietveld pattern of MS1 (d). The most visible peaks of La_2_O_3_ are marked with an asterisk.

**Table 1:** Summarized Values from the Density Measurements and Results from Rietveld Refinement

sample	measured density/g cm^–3^	calc. density/g cm^–3^	relative density/g cm^–3^	lattice parameter a/Å	atomic size difference *δ*/%
ref (DS)	5.478 ± 0.004	5.747	95.3	12.8718(4)	5.56
DS1	5.484 ± 0.004	5.840	93.9	12.8657(1)	5.98
DS2	5.576 ± 0.006	5.787	96.4	12.9012(2)	7.47
DS3	5.03 ± 0.02	5.502	91.4	12.9111(2)	7.47
DS4	5.10 ± 0.03	5.511	92.3	12.9105(1)	5.08
MS1	5.792 ± 0.004	5.842	99.1	12.8643(2)	5.98
MS2	5.912 ± 0.004	5.948	99.4	12.9093(1)	7.47
MS3	5.68 ± 0.02	5.749	98.9	12.9143(2)	7.47
MS4	5.337 ± 0.004	5.521	96.7	12.9020(3)	5.08

The density analysis reveals an increase of the density with a relative density close to 100% for the samples synthesized by the mono-step reaction (Table 1). Samples synthesized by the dual-step reaction achieved relative densities of around 92%. This increase in density with using a single heating program is in accordance with the tendencies observed by Xu et al.^33^ By eliminating a second milling step, the already formed grain connections do not get broken up again. The reformation of sinter necks during heat treatments takes time, which reduces the available time for the sample to densify.

The calculated values for δ are listed in Table 1 for the targeted compositions and Li_6_La_3_Zr_0.5_Nb_0.5_Ta_0.5_Hf_0.5_O_12_ as a reference (ref). The values for the new compositions are close to the reference compound whose successful synthesis was described in the literature.^22^ The ability to form single-phase garnets of the investigated systems is therefore supported by theoretical considerations. However, all these values are also above the threshold of around 5% reported for pyrochlore systems.^26^ This suggests that the garnet system can accommodate cations with a larger size difference than pyrochlores while remaining single phase.

After the phase purity of the samples is confirmed and the effect of the constituent elements on lattice parameters is studied, it is important to understand the elemental distribution and local morphology. The formation of two separate cubic garnets with differences in composition might not be detectable in XRD measurements as the lattice parameters might be too close or the amount of one phase in the sample is too low to be detected. SEM micrographs in Figure 2 show the morphology and grain distribution of the samples synthesized by the mono-step and dual-step methods. The images reveal an increase in grain size from dual-step to mono-step method. This observation fits the higher relative densities of the samples. In comparison, the images of the dual-step samples reveal a porous network of grains with large gaps between the grain clusters. This indicates incomplete sintering of the samples. There was, however, no clear distinction in morphology between the samples synthesized by the same method. Thus, to better highlight the surface features like grain boundaries, more close-up pictures for MS1 and MS2 are shown. The morphological analysis also revealed small particles on the surface that differ in appearance from those in the surrounding material. This most likely is a layer of Li_2_CO_3_ on samples that had some exposure to the air. This is supported by comparing fresh samples, which show minimal to no contamination, compared to samples with more exposure to air. The latter shows more of these particles. The island-like distribution across the exposed samples is in accordance with what is reported in the literature after extensive exposure.^36,37^ It is therefore important to thoroughly remove the surface of the samples before the electrochemical measurements to avoid the influence of impurities on the results.

**Figure 2:**
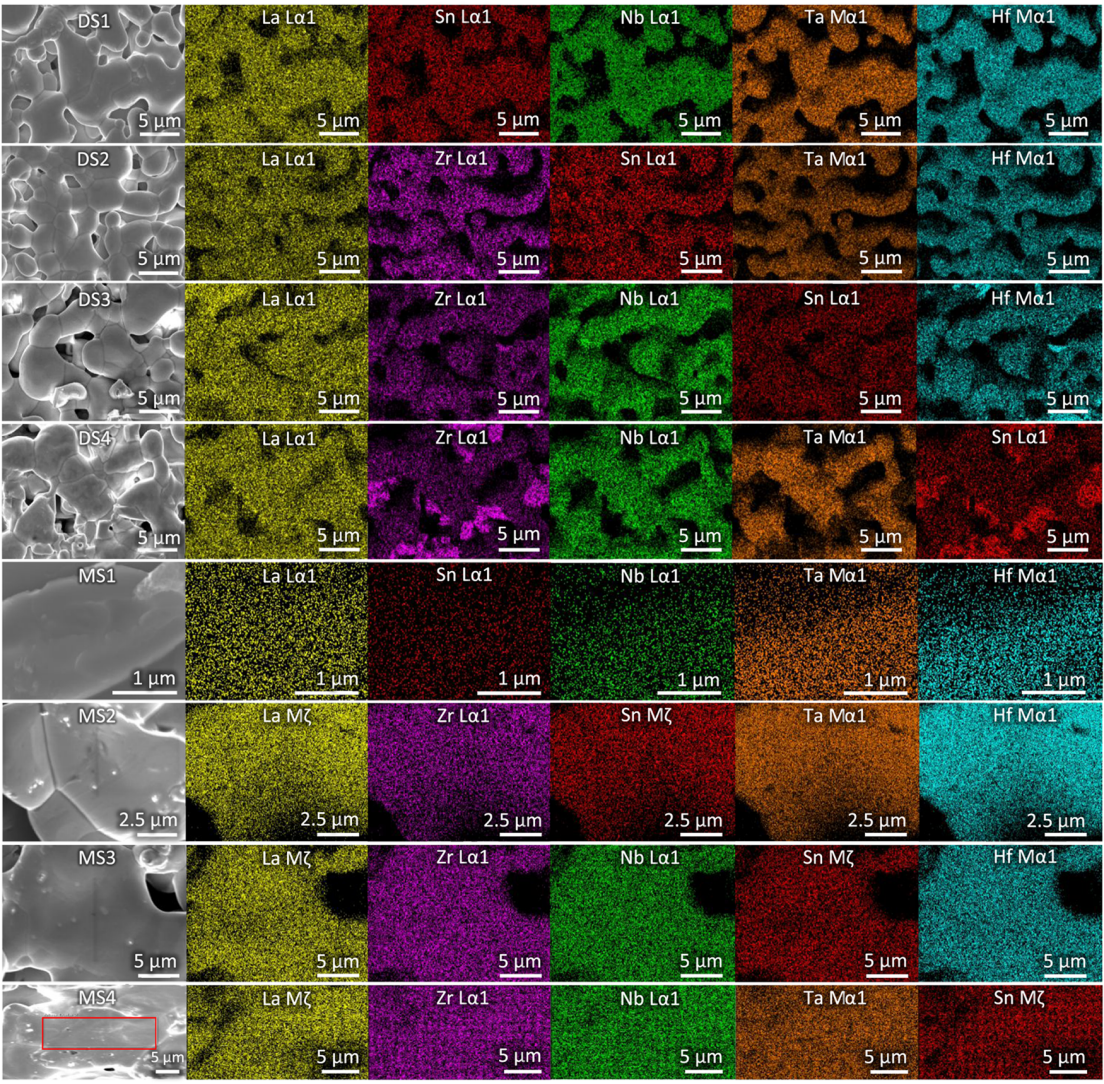
Elemental mappings of the dual-step samples DS1–4 and the mono-step samples MS1–4 from top to bottom. The red box represents the measured area.

The elemental composition of the samples was analyzed with energy-dispersive X-ray spectroscopy (EDS) to create maps of the elemental distribution (Figure 2). The resulting element ratio (Figure 3) fits the targeted nominal compositions of Li_6_La_3_Sn_0.5_Nb_0.5_Ta_0.5_Hf_0.5_O_12_, Li_6.5_La_3_Zr_0.5_Sn_0.5_Ta_0.5_Hf_0.5_O_12_, Li_6.5_La_3_Zr_0.5_Nb_0.5_Sn_0.5_Hf_0.5_O_12_, and Li_6_La_3_Zr_0.5_Nb_0.5_Ta_0.5_Sn_0.5_O_12_ closely. Due to being a very light element and its electron configuration, the lithium content could not be quantified. Oxygen was also excluded from quantification to reduce error margins. The heavy elements in the sample are easier to detect with EDS, but due to it being a rather insensitive method, slight deviations in the quantification results are to be expected. Based on Figure 2, there seems to be a homogeneous distribution of the constituent elements, with DS3 showing slight clustering of Zr and Hf, with the quantification showing an elevated Nb content, and DS4 with some clusters of Sn and Zr. These inhomogeneities might be attributed to slight differences in synthesis conditions (volatilization of lithium, absorbed water). Homogeneous distribution is the foundation of the “high-entropy” approach as it is necessary that all elements are incorporated statistically in the same crystal lattice to enable the configurational entropy effect. The results from SEM measurements in combination with the data from the XRD pattern indicate that single-phase garnet structures with all elements incorporated were synthesized via the mono-step approach.

**Figure 3:**
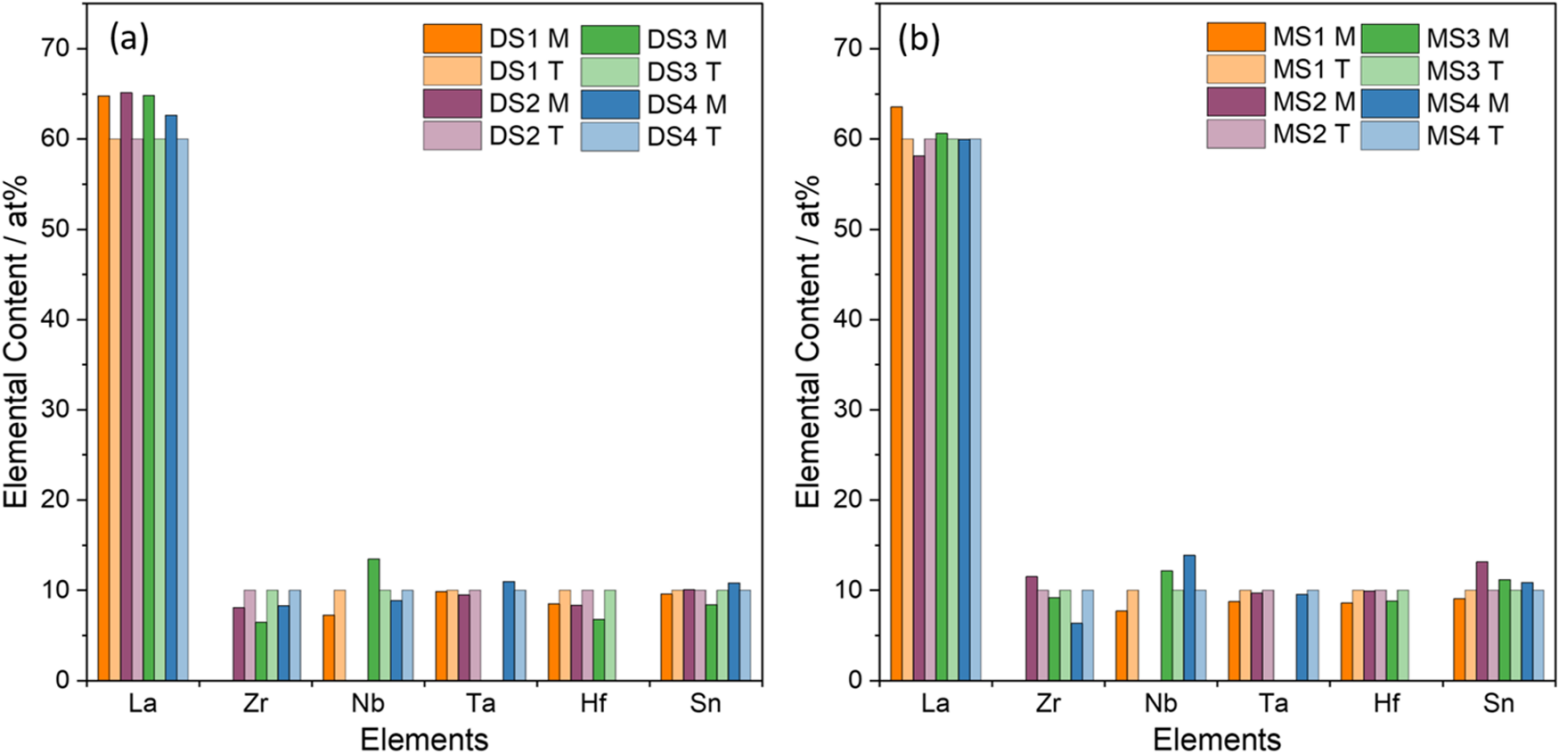
Results of the quantitative EDS analysis of DS1–4 (a) and MS1–4 (b) with measured values (M) and theoretical values (T).

### 3.2 Characterization of Electrochemical Properties

The structure analysis and determination of the chemical composition and distribution enabled a good framework for further investigation. Based on these results, the influence of the structure on the ionic transport was studied via electrochemical impedance spectroscopy (EIS). Figure 5 shows the results from the electrochemical characterization with AC room temperature (25 °C) and Arrhenius measurements between −40 and 40 °C to determine ionic conductivity and the activation energy, respectively. The impedance plots for the remaining temperatures are given in Figures S4–S23 in SI. All samples show a well-defined semicircle at high frequencies, representing the bulk conduction of the characterized materials. The long tail at low frequencies represents the polarization of the material due to the ion-blocking effect of gold electrodes. Three samples (DS2, MS2, MS3) also show a deviation from the remaining samples in the middle frequency range, which originates from an additional second semicircle representing the charge transfer across grain boundaries. The semicircles within the impedance spectrum are linked to the respective processes through their capacitance *C* and characteristic relaxation time τ. With a capacitance of around 10^–11^ F and a relaxation time of 0.5 μs, the high-frequency semicircle can easily be identified to represent the bulk ionic contribution. The second separate semicircle of some samples with a capacity of around 10^–8^ F and a relaxation time of 30 μs can then be linked to grain boundary conduction, which fits well to literature observations.^38,39^ All respective calculated parameters are listed in Table S1 in the SI. The other samples did not show a clear differentiation between both semicircles in the impedance plots (Figure 5), so only the total conductivity values are given. The equivalent circuits used to fit the data are shown as exemplary in Figure 4. The measured values for the ionic conductivities and calculated activation energies from the impedance spectra are given in Table 2. When comparing the mono-step (MS) samples, a trend in ionic conductivities can be observed. The samples where a pentavalent ion (Ta^5+^/Nb^5+^) has been replaced by Sn^4+^ are about two times more conductive than the samples where both pentavalent cations are present, increasing from ∼9 × 10^–5^ to ∼2 × 10^–4^ S cm^–1^. One reason for this behavior could be the higher lithium content of 6.5 vs 6 per formula unit of samples without and with Ta^5+^/Nb^5+^, respectively. As reported in the literature, a lithium content of 6.5 per formula unit is the amount with the highest carrier concentration,^40^ which should lead to the highest ionic conductivity. For the dual-step samples, DS3 also has a higher ionic conductivity as compared to DS1 and DS4, although the effect is smaller compared to the mono-step samples. Possibly, due to the lower density, the associated error diminishes the positive effect of a higher lithium content in the sample. DS2 seems to be an outlier with the lowest conductivity across all measured samples, and it is also the only sample from that group where a second semicircle could be resolved. When comparing the two synthesis methods, the mono-step samples showed higher conductivity for the cases with the highest lithium content while having lower conductivities for the other case. Possible explanations for the described deviations from the expected trend are plenty as an interlaboratory study conducted by multiple groups suggests.^41^

**Figure 4:**
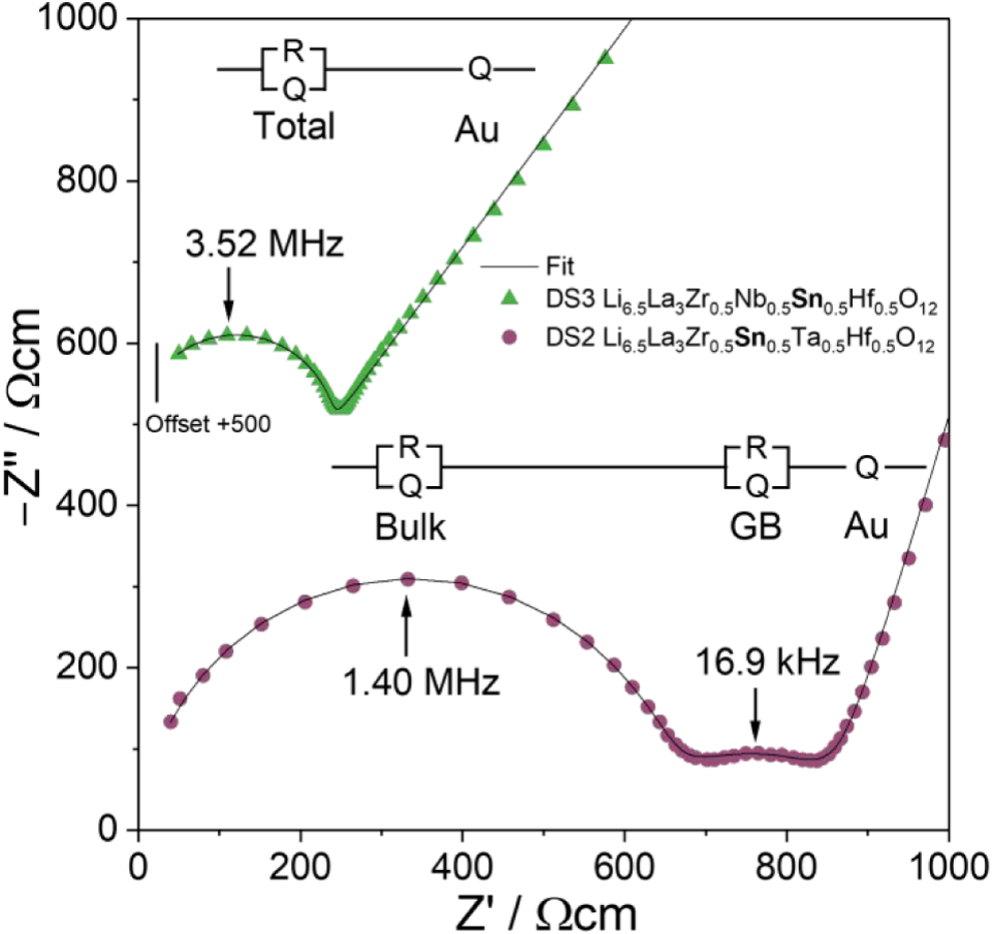
Impedance spectra of DS2 (bottom) and DS3 (top) with their respective equivalent circuits and peak frequencies.

**Figure 5:**
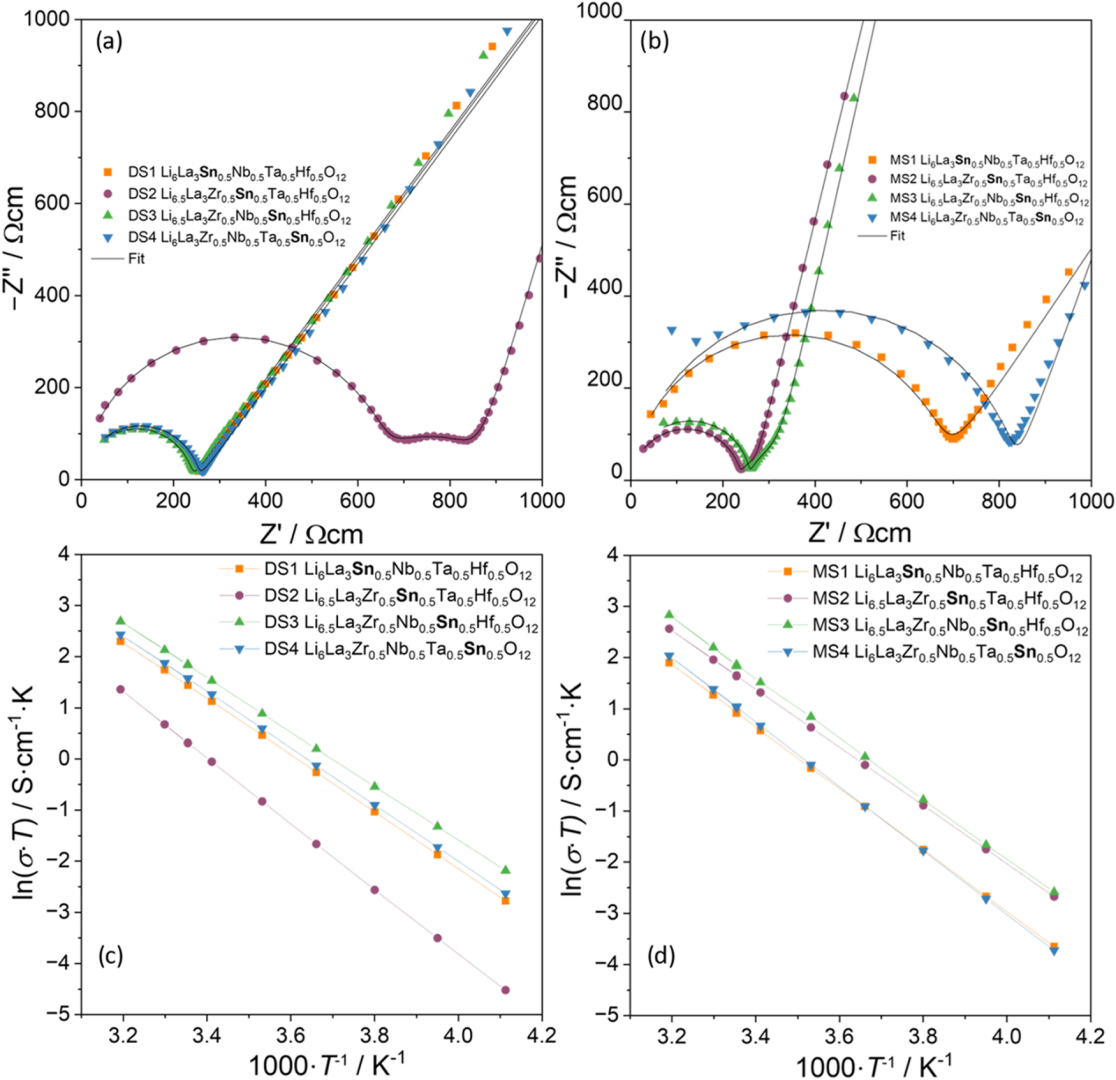
Normalized impedance spectra for the dual- (a) and mono-step (b) samples as well as their respective Arrhenius plots to determine total ionic conductivity­(c and d).

**Table 2:** Measured Ionic Conductivity and Calculated Activation Energy for the Mono- and Dual-Step Samples

sample	total Ionic conductivity (25 °C)/S cm^–1^	bulk activation energy *E*_A_/eV	GB activation energy *E*_A_/eV	total activation energy *E*_A_/eV
DS1	1.41 × 10^–4^			0.46
DS2	4.57 × 10^–5^	0.547	0.567	
DS3	2.10 × 10^–4^			0.48
DS4	1.62 × 10^–4^			0.48
MS1	8.54 × 10^–5^			0.52
MS2	1.75 × 10^–4^	0.484	0.541	
MS3	2.12 × 10^–4^	0.487	0.580	
MS4	9.38 × 10^–5^			0.54

Since the reported values are close in order of magnitude when comparing a DS sample to their respective MS sample, slight differences in composition might already impact the conductivity. Another factor is the high volatility of lithium oxide, making accurate targeting of lithium content difficult. Due to less time being spent on heating or cooling when using the mono-step approach, a lower lithium loss is expected, which could improve the conductivity. This, together with the higher density of MS samples, could explain their enhanced ionic conductivity. Furthermore, the impurity phase (La_2_O_3_) detected in DS2 and aggregation of Zr/Hf and Sn/Zr in DS3 and DS4, respectively, have an impact on the measured ionic conductivity for the dual-step samples. The calculated activation energies follow the observations in the conductivity. Samples with lower conductivity have higher activation energies, although the differences are rather small. In total, the activation energies in the range of 0.50 eV are rather high compared to Al-doped LLZO with reported activation energy of 0.34 eV and ionic conductivity of 4 × 10^–4^ S cm^–1^.^42^ A possible factor could be the lattice parameter of the investigated samples, which will be discussed in section 3.3.

### 3.3 Structural Influence on Ionic Transport

After establishing the outline of the structural and electrochemical properties of the synthesized material, it is important to discuss them in a combined context. Due to the compositional uncertainties in the dual-step samples, only the mono-step samples are discussed in this section. The correlation between the activation energy and ionic conductivity can be deduced from Figure 6. An increase in activation energy leads to a reduction in ionic conductivity according to the expectations as higher activation energies mean less mobility of the ion carriers. Earlier, it was established that the determined lattice parameters of the tin garnets are smaller than Al-doped cubic LLZO, while a reduction in ionic conductivity was also observed. One reason could be a decrease in the size of the Li conduction channels due to the reduction in lattice parameters compared to LLZO, thus increasing the migration resistance, which in turn increases the activation energy *E*_a_.^6,31^ To study whether this is the case, the activation energy and ionic conductivity for the MS samples have been plotted against the lattice parameter in Figure 6. The data shows an increase of the ionic conductivity by factor two from the sample with the lowest to the highest lattice parameter. While the total increase in ionic conductivity is also very small, similar changes in this lattice parameter range were previously reported by Zeier et al.^31^ Still, these small changes make the derived correlation prone to errors from measurement, sample inhomogeneities, and derivation in the calculated lattice parameters. Also, since two different Li contents in the formula were investigated, it is difficult to determine how these different factors interact with each other as well. However, a comparison of the investigated samples to the values of Li_6.5_La_3_Sn_1.5_Ta_0.5_O_12_ reported in literature^32^ (1.9 × 10^–4^ S cm^–1^, 0.451 eV) seems to suggest a correlation between elevated activation energies and tin. Li_6_La_3_Zr_0.5_Nb_0.5_Ta_0.5_Hf_0.5_O_12_ sintered for 12 h^22^ shows a lower conductivity than would be anticipated based on lattice parameters. No activation energy was provided for this sample. Future studies are necessary to determine, for example, whether the optimization of lattice parameters or lithium content is more beneficial toward higher ionic conductivities and in which way tin might influence activation energies. It is possible that an increase in the lattice parameter and thus an increase in ion mobility is offset by reducing the total number of available charge carriers.

**Figure 6:**
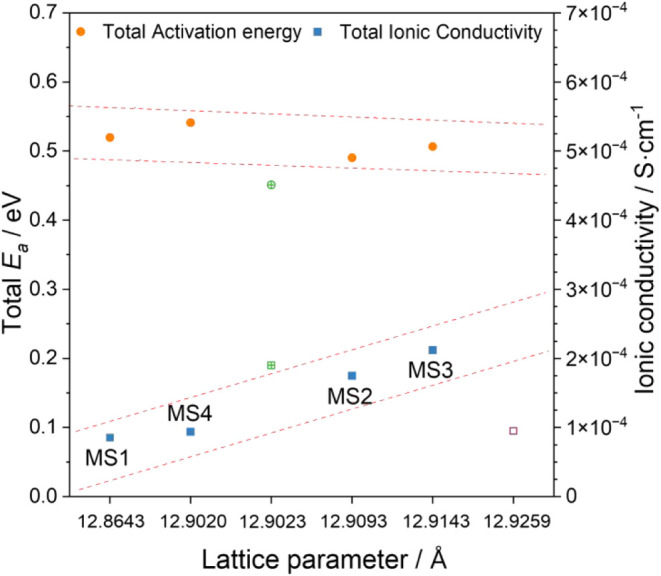
Plot of the ionic conductivity (blue) and activation energy (orange) against the calculated lattice parameter of the mono-step samples. The green data points (crossed) represent Li_6.5_La_3_Sn_1.5_Ta_0.5_O_12_ ^32^ and the purple data point (hollow) Li_6_La_3_Zr_0.5_Nb_0.5_Ta_0.5_Hf_0.5_O_12_ after 12 h sintering.^22^

## 4 Conclusions

Four different cubic garnets with the general formula Li_6_La_3_ **Sn**_0.5_Nb_0.5_Ta_0.5_Hf_0.5_O_12_, Li_6.5_La_3_Zr_0.5_ **Sn**_0.5_Ta_0.5_Hf_0.5_O_12_, Li_6.5_La_3_Zr_0.5_Nb_0.5_ **Sn**_0.5_Hf_0.5_O_12_, and Li_6_La_3_Zr_0.5_Nb_0.5_Ta_0.5_ **Sn**_0.5_O_12_ have been synthesized each by two different solid-state synthesis approaches. The samples have been characterized by XRD with further Rietveld analysis, SEM, and EIS measurements. Additionally, the densities of the samples have been determined via gas pycnometry. The XRD measurements revealed phase-pure samples for the mono-step approach, while the density measurements showed an increase in density for these samples. The Rietveld refinement revealed a reduction in lattice parameters compared to those of cubic LLZO, likely due to the replacement of Zr^4+^ with smaller ions. The information from XRD analysis together with the calculated atomic size factors indicates a successful incorporation of the different metal cations on the B site. EDS proved in addition that the metal cations, in general, are evenly distributed throughout the samples. Ionic conductivities derived from electrochemical impedance spectroscopy ranged between 4.57 × 10^–5^ and 2.12 × 10^–4^ S cm^–1^ with activation energies for the bulk transport between 0.46–0.58 eV. These values are slightly lower than for cubic Al-doped Li_7_La_3_Zr_2_O_12_ reported in the literature. A study of a possible effect of the change in lattice parameters on ionic conductivity did not lead to a concise result. Further experiments are necessary to validate literature data that suggest a positive correlation between lattice parameters and ionic conductivity. The prescreening of element combinations via atomic size factor δ proved useful in the researched systems; however, further studies need to be made to determine the limits and applicability on the garnet system. One of the key advantages of Li-garnets is their compatibility with lithium metal. However, by substitution of LLZO with multiple elements, these also change the redox potential of the material and might change the compatibility. For this reason, it is also necessary for further studies to explore the impact of different element combinations on the interface interactions between high-entropy garnets and lithium metal, regarding future applications of this material class.

## Supplementary Material



## Data Availability

The data underlying this study is available in the published article and its Supporting Information.

## Notes

Additional figures with impedance spectra, Rietveld refinement, and table with electrochemical data (PDF)

## Abbreviations

SEM: scanning electron microscope
HEA: high-entropy alloy
XRD: X-ray diffraction
EIS: electrochemical impedance spectroscopy
EDS: energy-dispersive X-ray spectroscopy
LLZO: Li_7_La_3_Zr_2_O_12_
FCC: face-centered cubic
BCC: body-centered cubic
DS: dual-step
MS: mono-step
wt %: weight percent
