# Supplementary material for: Structural Analysis of Tin-Substituted High-Entropy Li-Garnet Electrolytes for Solid-State Batteries
Source: ACS Org Inorg Au. 2025 Apr 30;5(3):211–20. doi: 10.1021/acsorginorgau.5c00021 (PMC12142435; doi:10.1021/acsorginorgau.5c00021)
Supplement: Supplementary file 1 [file gg5c00021_si_001.pdf]

Structural analysis of tin substituted high-entropy Li-garnet electrolytes for solid-state batteries

Benjamin Zimmermann[1], Till Fuchs[2,3], Johannes Westphal[1], Jürgen Janek[2,3], Maren Lepple[1,3]*

[1]Institute of Inorganic and Analytical Chemistry, Justus Liebig University Giessen, Heinrich-Buff-Ring 17, 35392 Giessen, Germany

[2]Institute of Physical Chemistry, Justus Liebig University Giessen, 35392 Giessen, Germany

[3]Center for Materials Research, Justus Liebig University Giessen, 35392 Giessen, Germany

Supporting Information

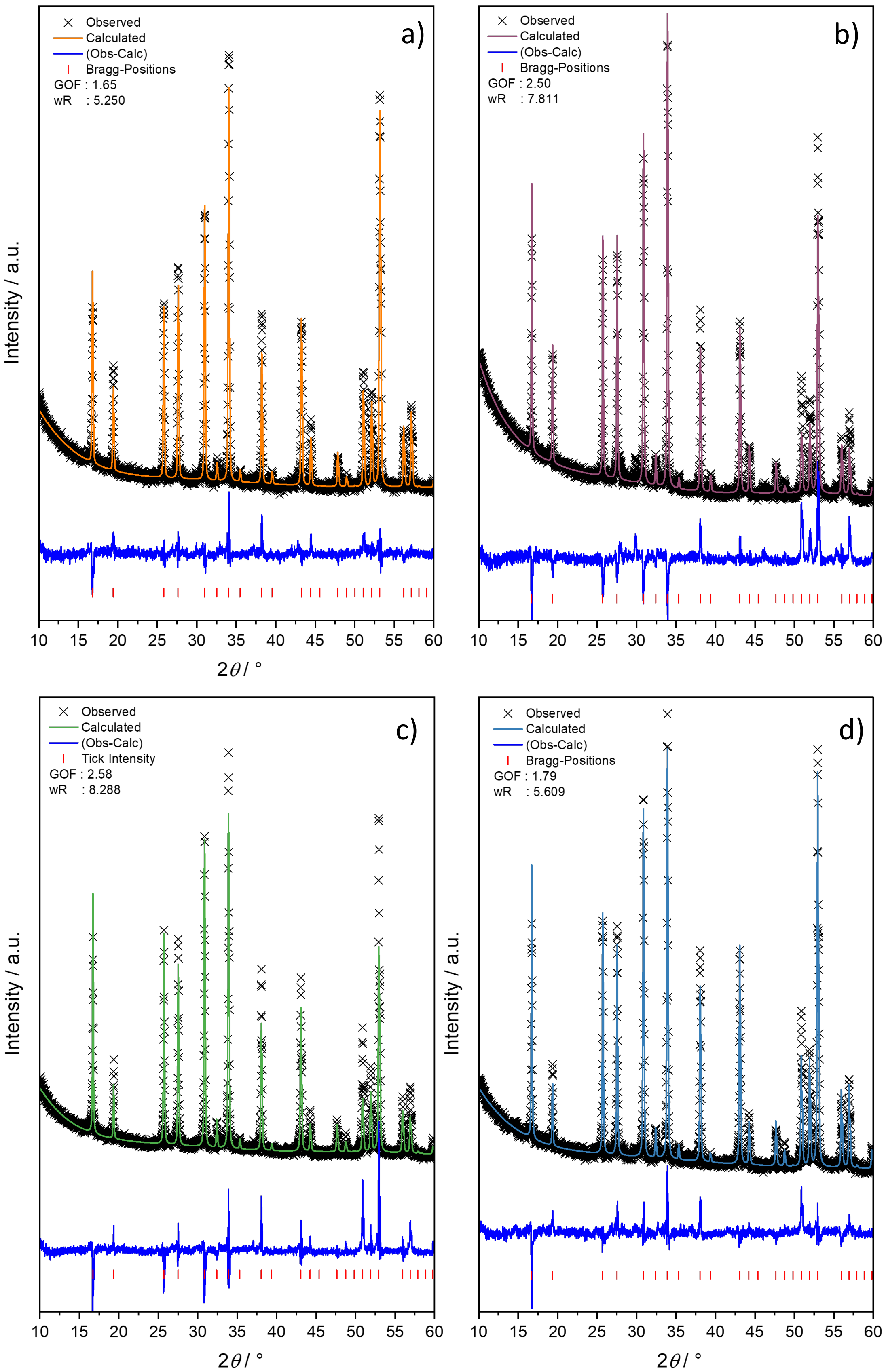

Figure 1. Graphs received from Rietveld refinement of DS1 (a), DS2 (b), DS3 (c) and DS4 (d).

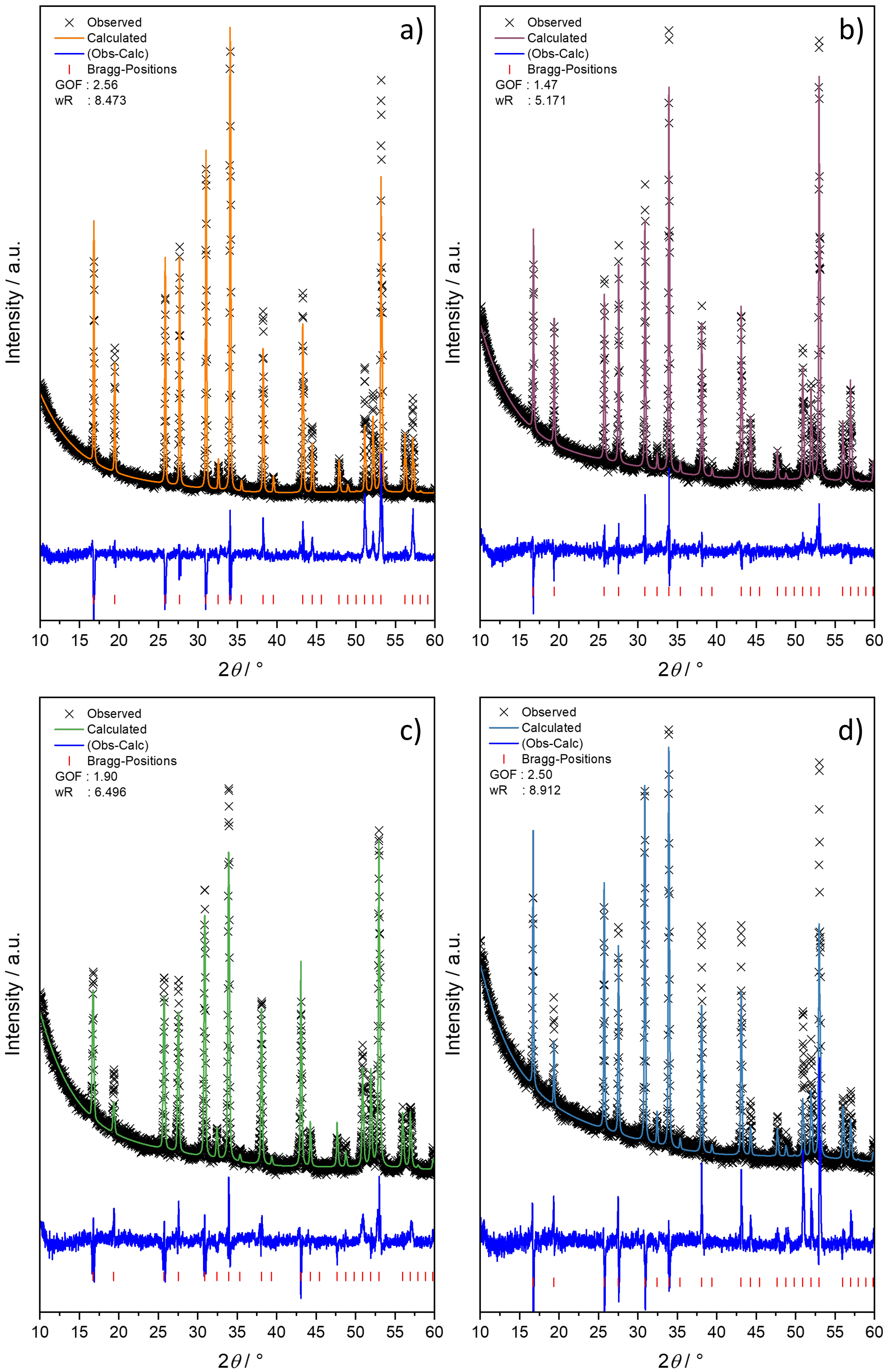

Figure 2. Graphs received from Rietveld refinement of MS1 (a), MS2 (b), MS3 (c) and MS4 (d).

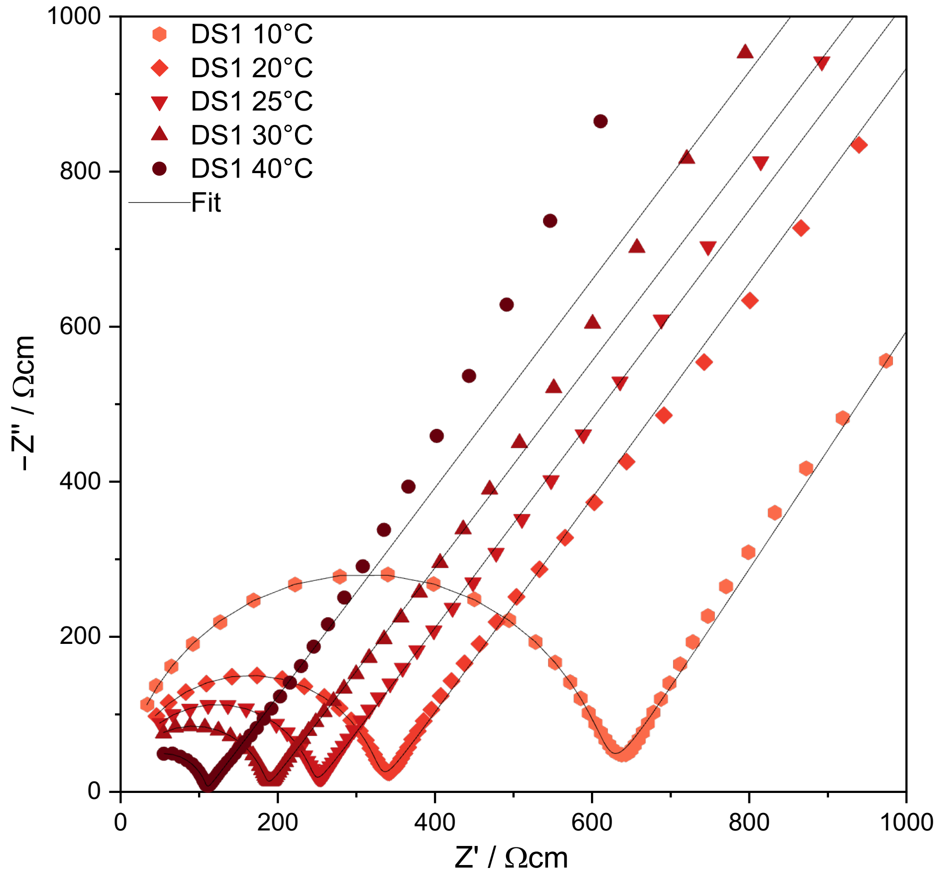

Figure 3. AC impedance spectrum of DS1 from 40°C to 10°C.

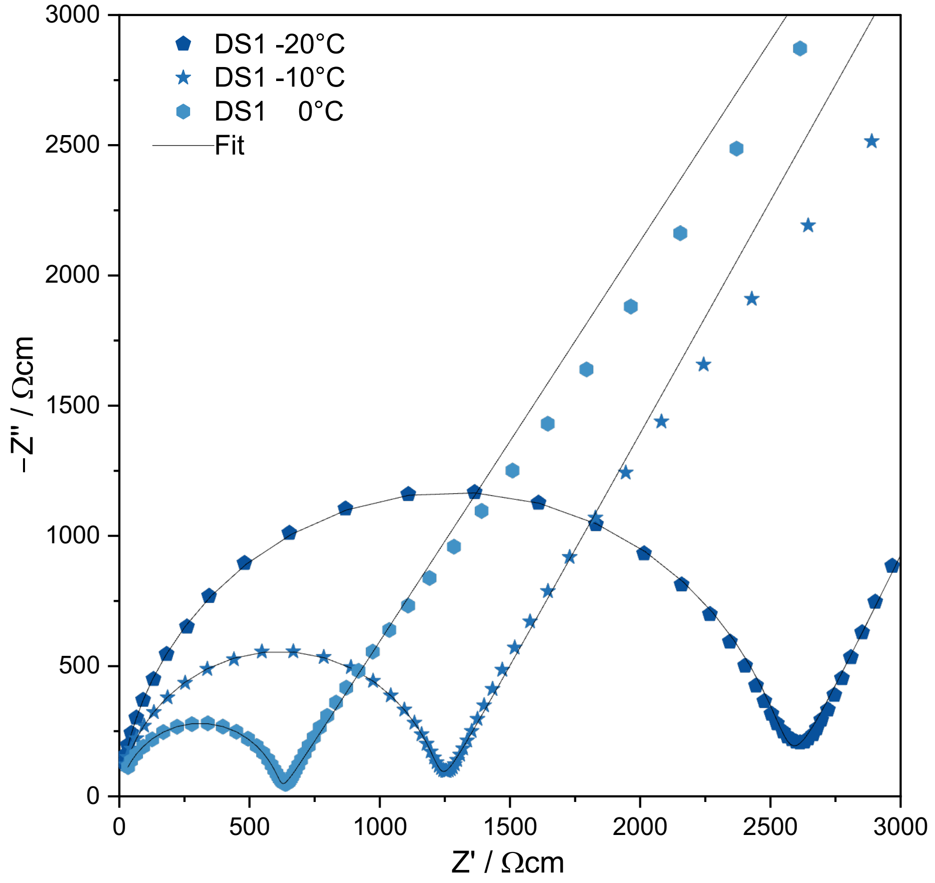

Figure 4. AC impedance spectrum of DS1 from 0°C to -20°C.

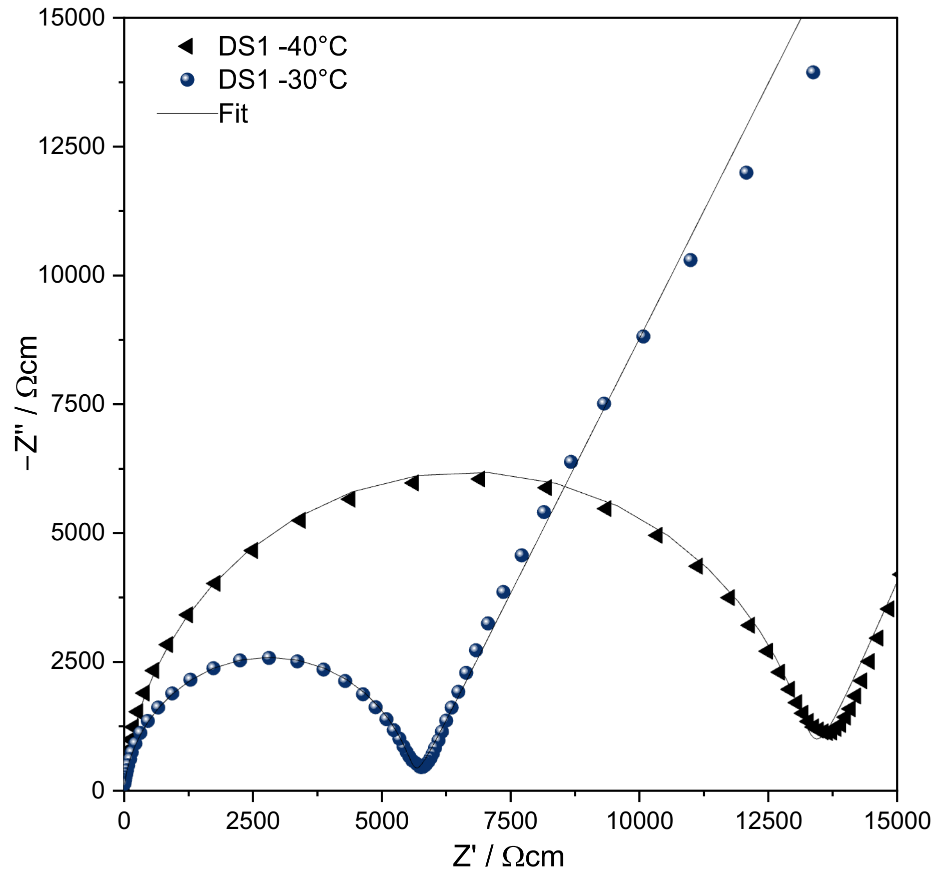

Figure 5. AC impedance spectrum of DS1 from -30°C to -40°C.

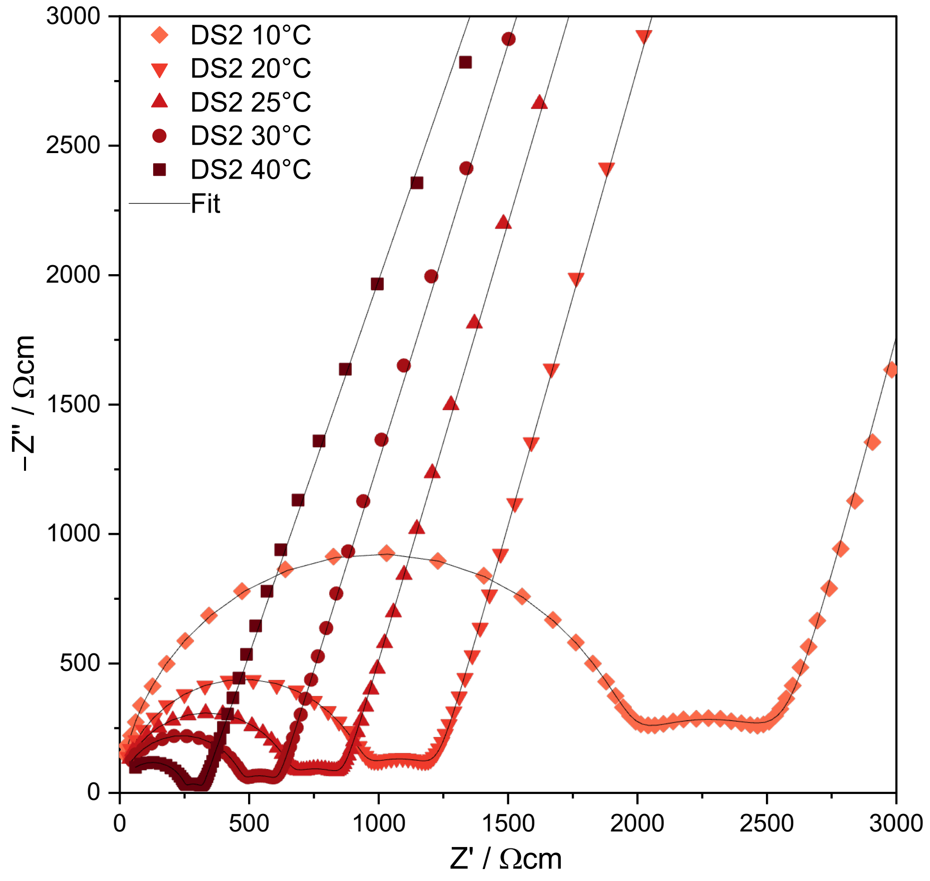

Figure 6. AC impedance spectrum of DS2 from 40°C to 10°C.

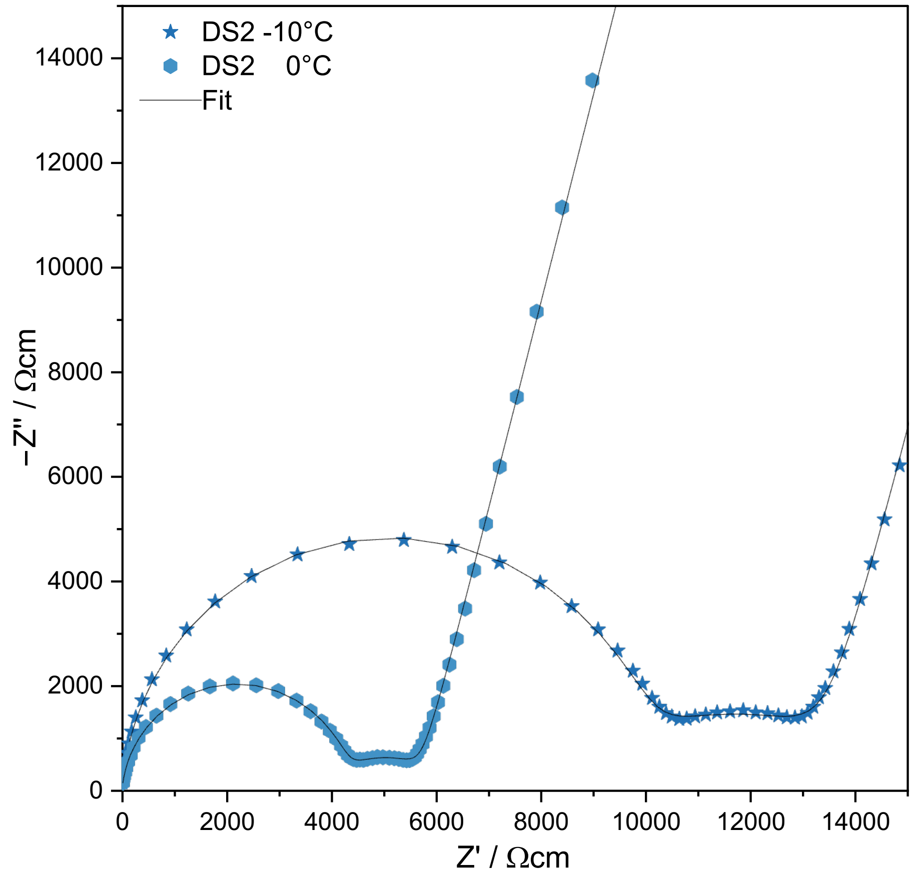

Figure 7. AC impedance spectrum of DS2 from 0°C to -10°C.

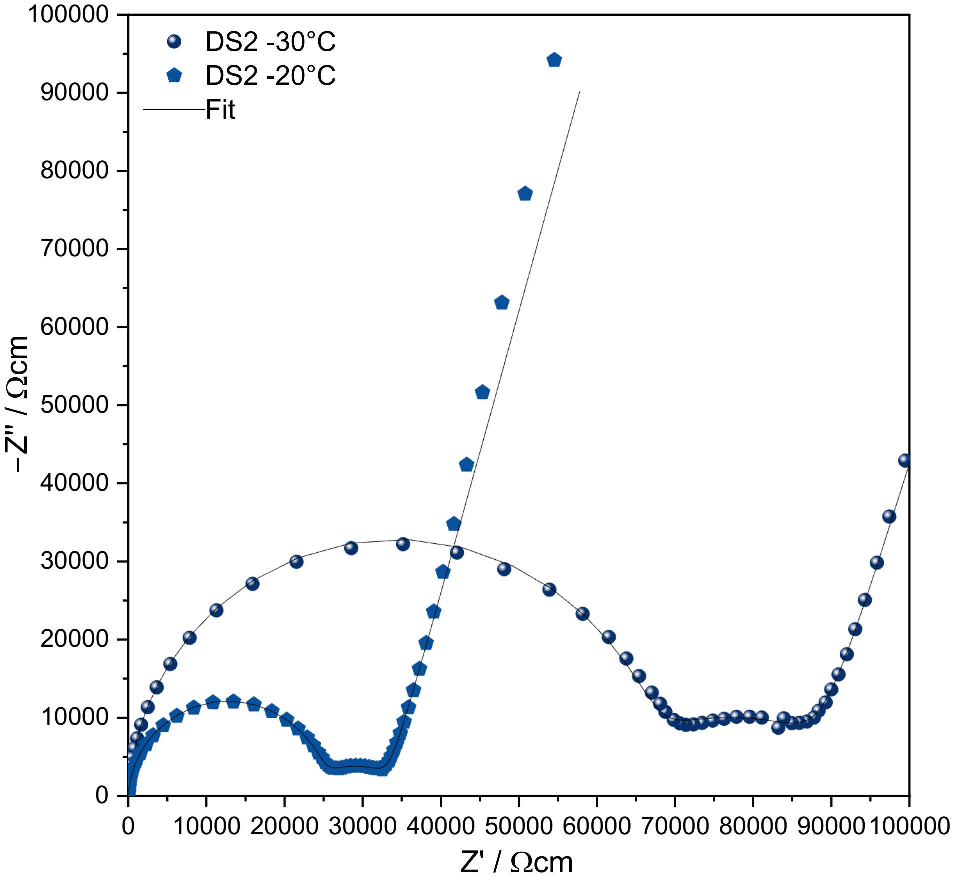

Figure 8. AC impedance spectrum of DS2 from -20°C to -30°C.

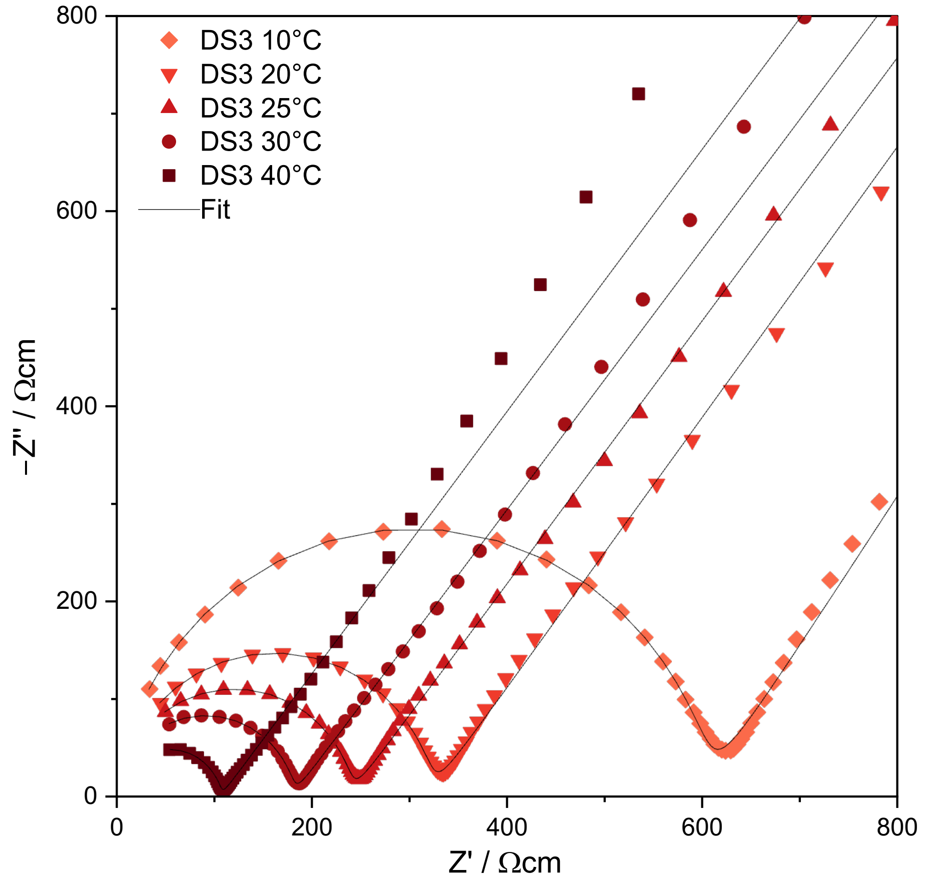

Figure 9. AC impedance spectrum of DS3 from 40°C to 10°C.

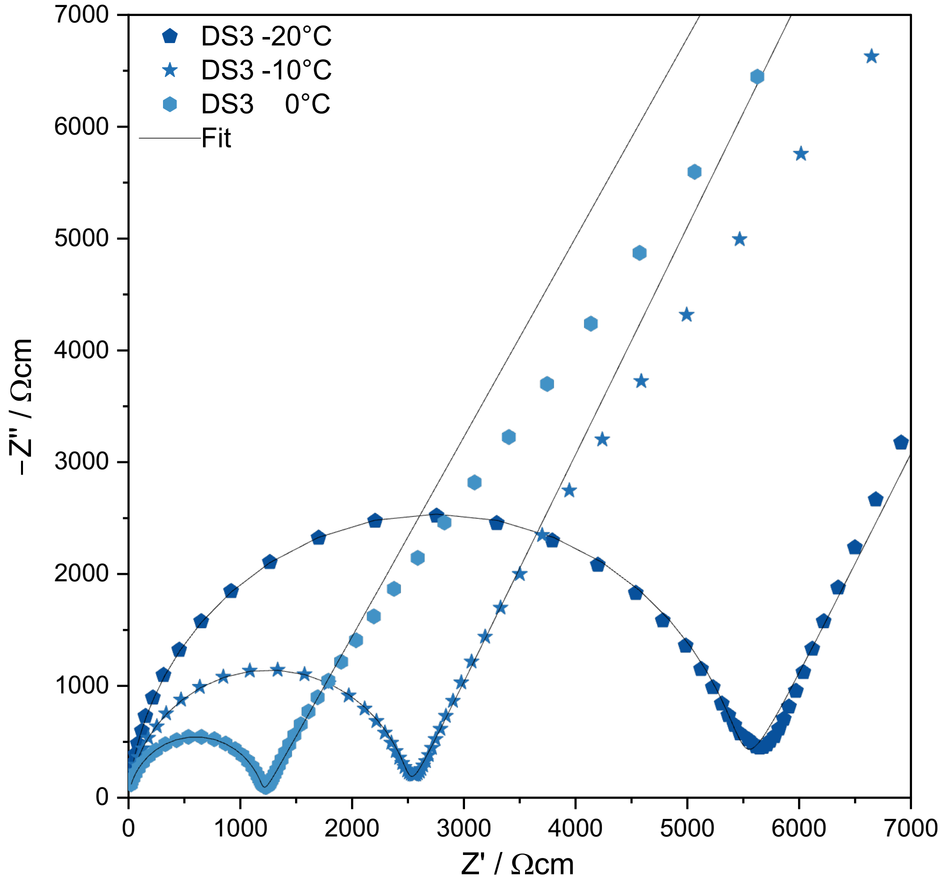

Figure 10. AC impedance spectrum of DS3 from 0°C to -20°C.

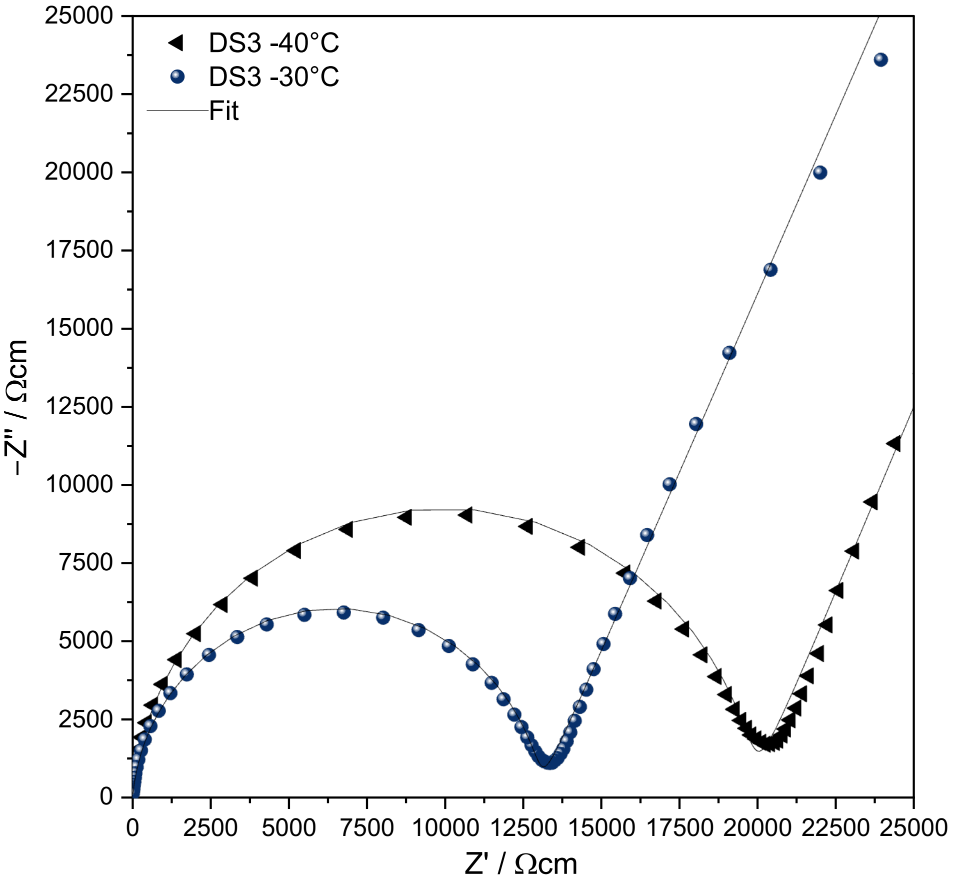

Figure 11. AC impedance spectrum of DS3 from -30°C to -40°C.

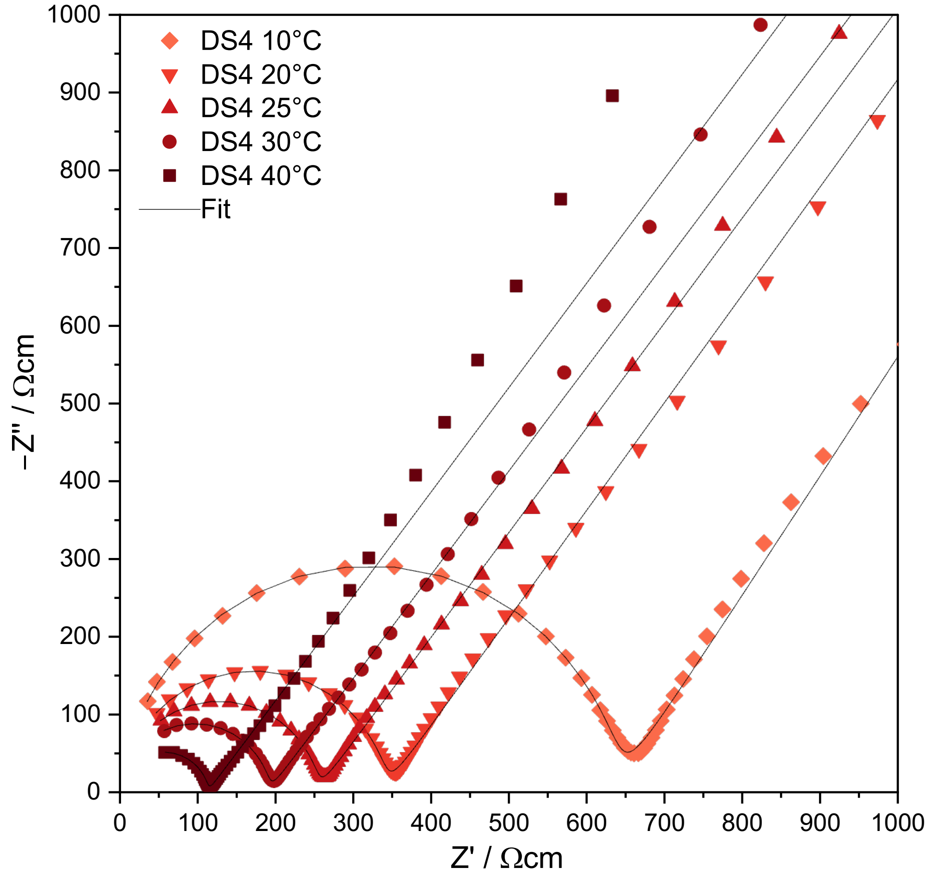

Figure 12. AC impedance spectrum of DS4 from 40°C to 10°C.

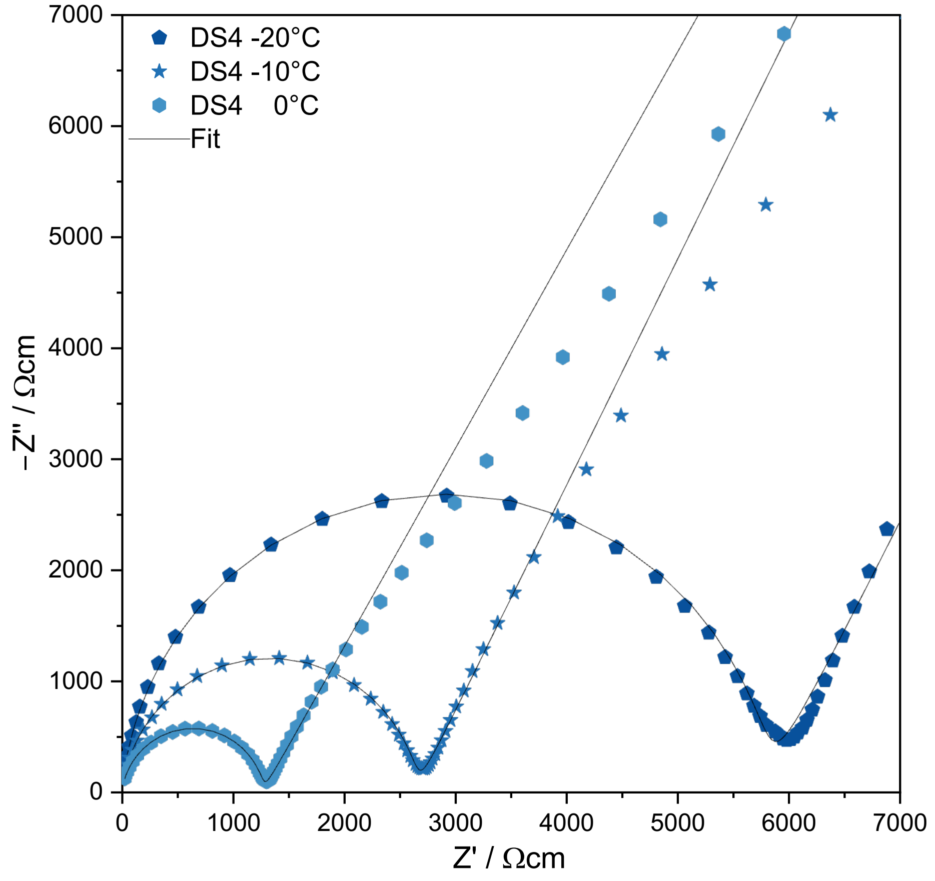

Figure 13. AC impedance spectrum of DS4 from 0°C to -20°C.

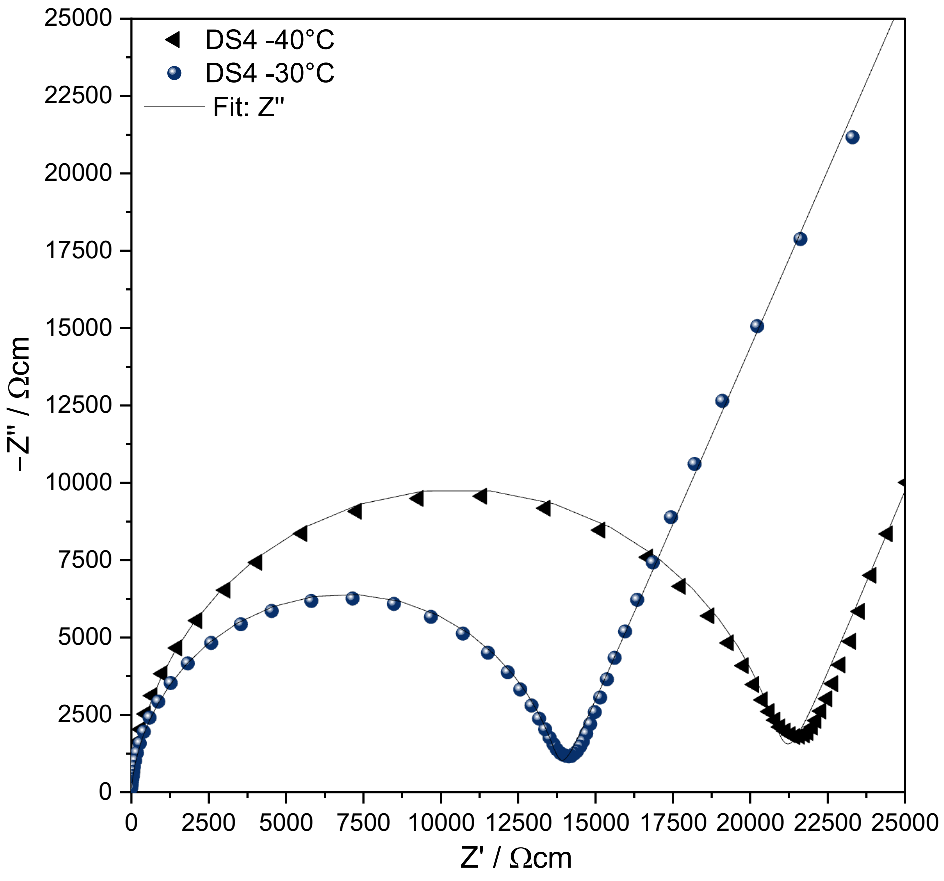

Figure 14. AC impedance spectrum of DS4 from -30°C to -40°C.

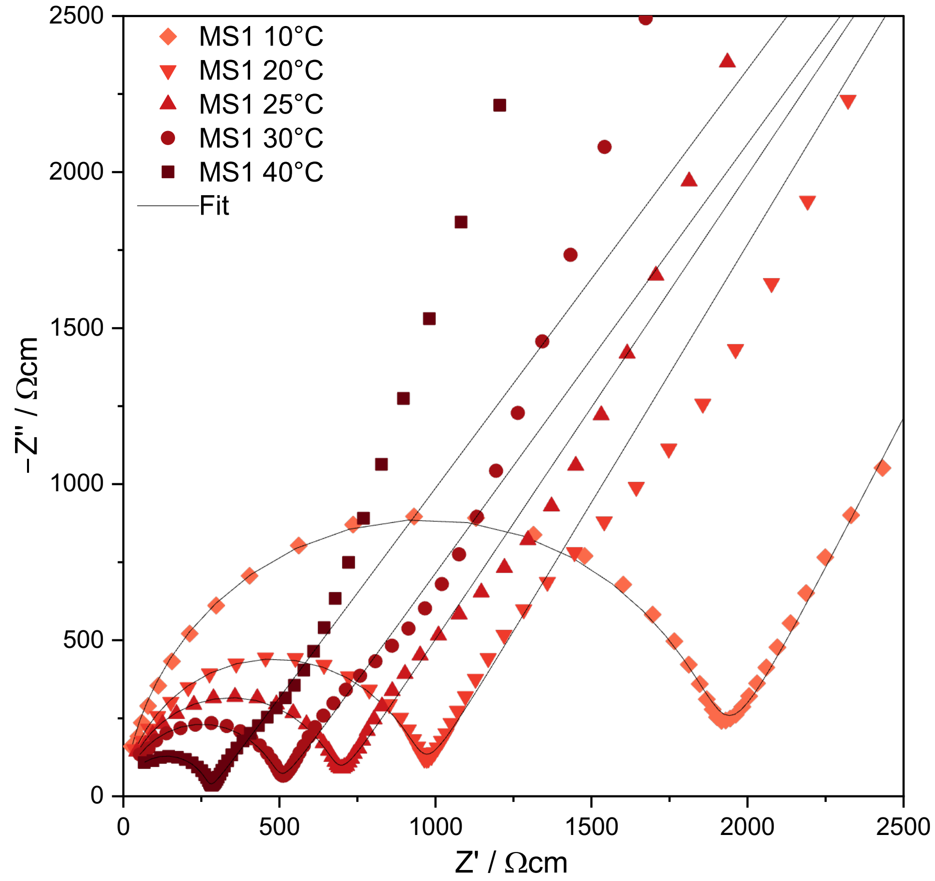

Figure 15. AC impedance spectrum of MS1 from 40°C to 10°C.

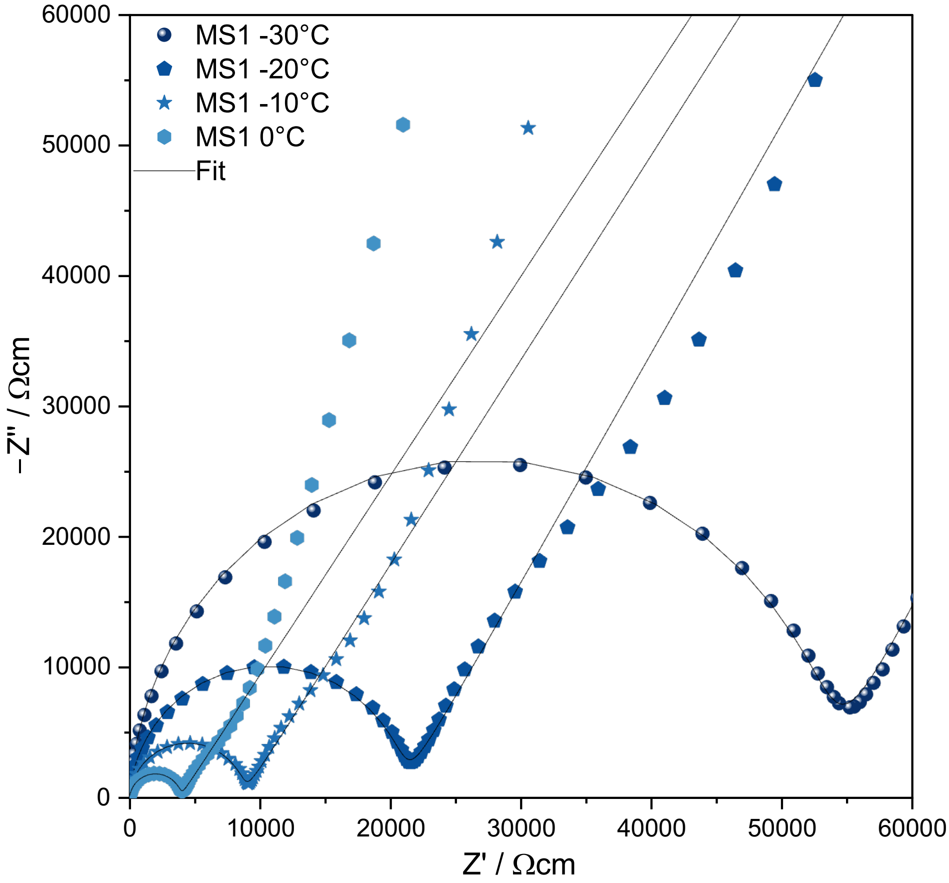

Figure 16. AC impedance spectrum of MS1 from 0°C to -30°C.

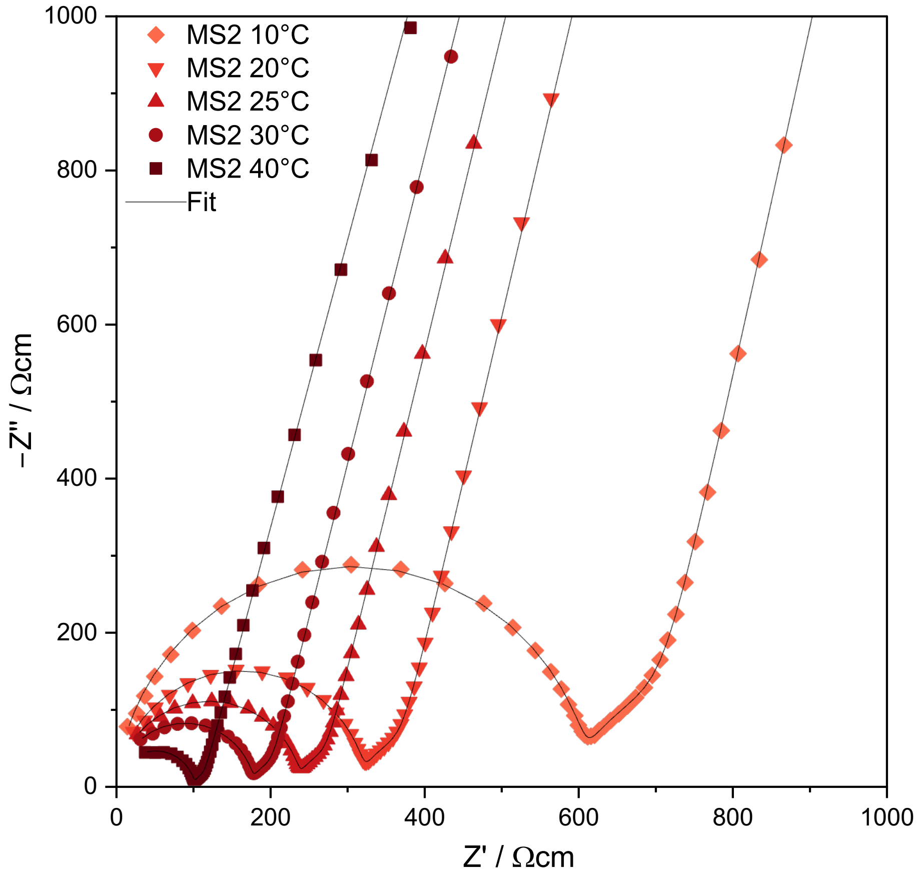

Figure 17. AC impedance spectrum of MS2 from 40°C to 10°C.

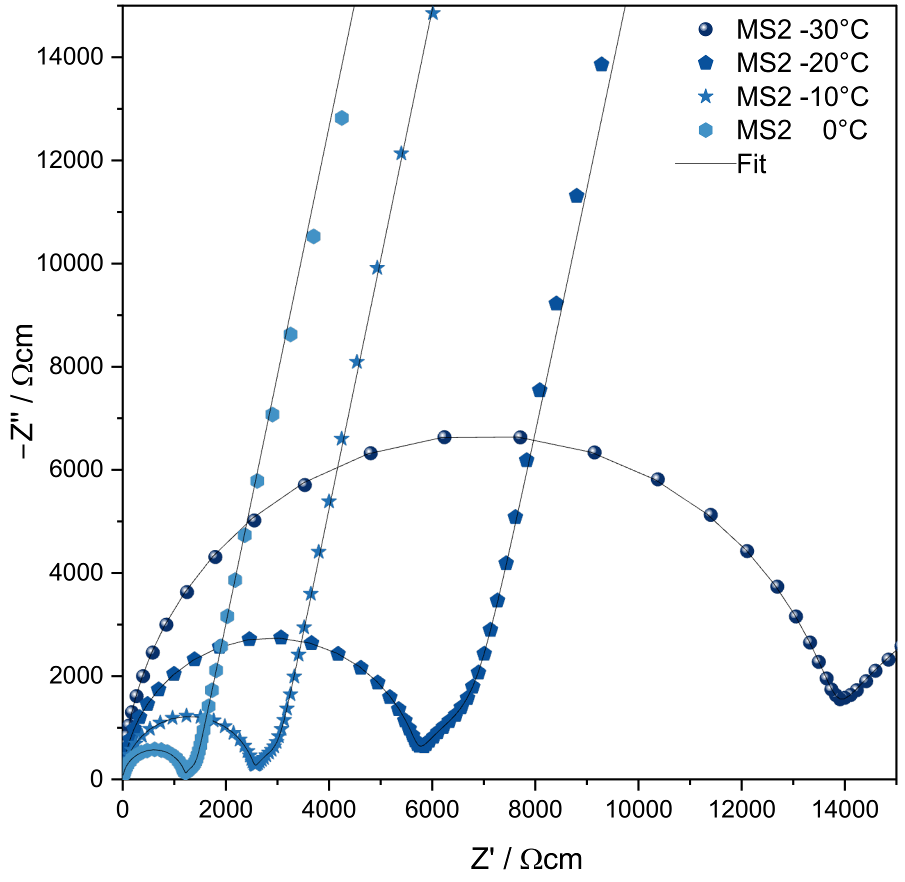

Figure 18. AC impedance spectrum of MS2 from 0°C to -30°C.

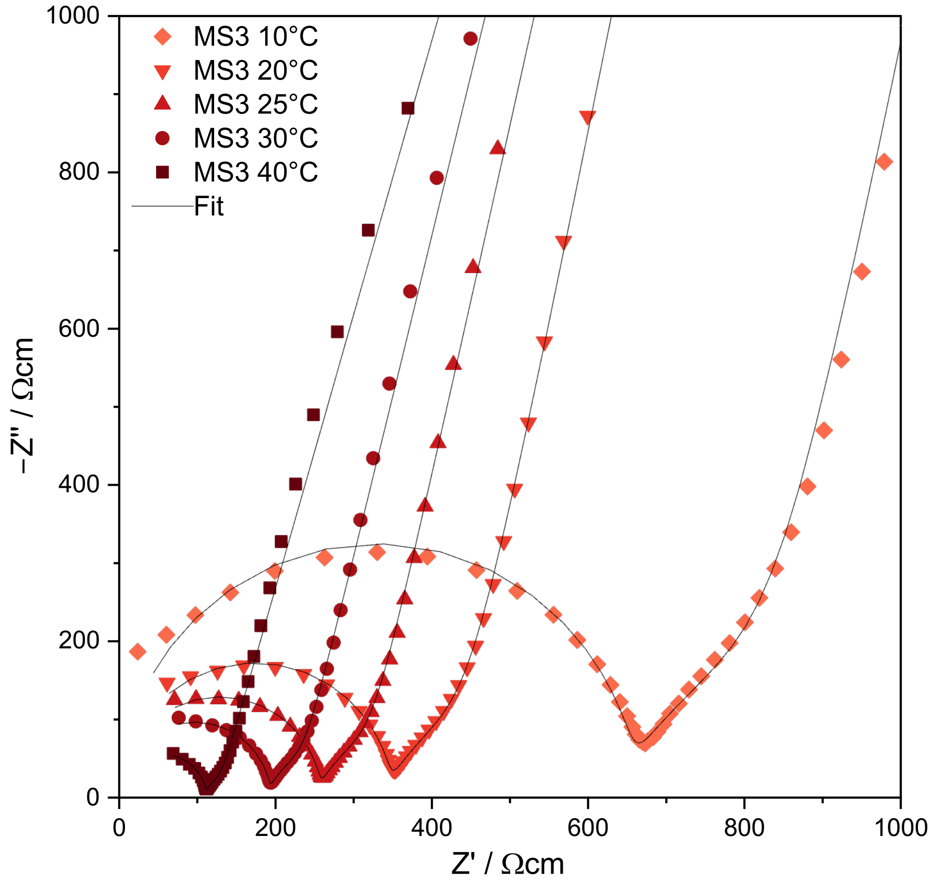

Figure 19. AC impedance spectrum of MS3 from 40°C to 10°C.

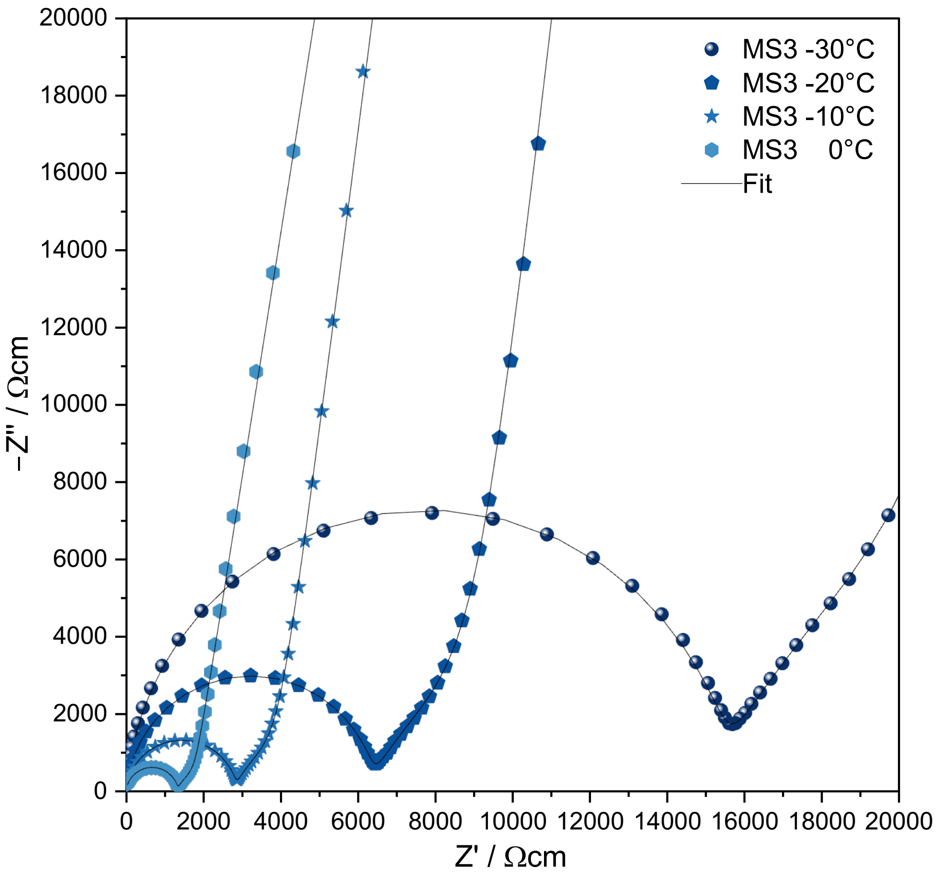

Figure 20. AC impedance spectrum of MS3 from 0°C to -30°C.

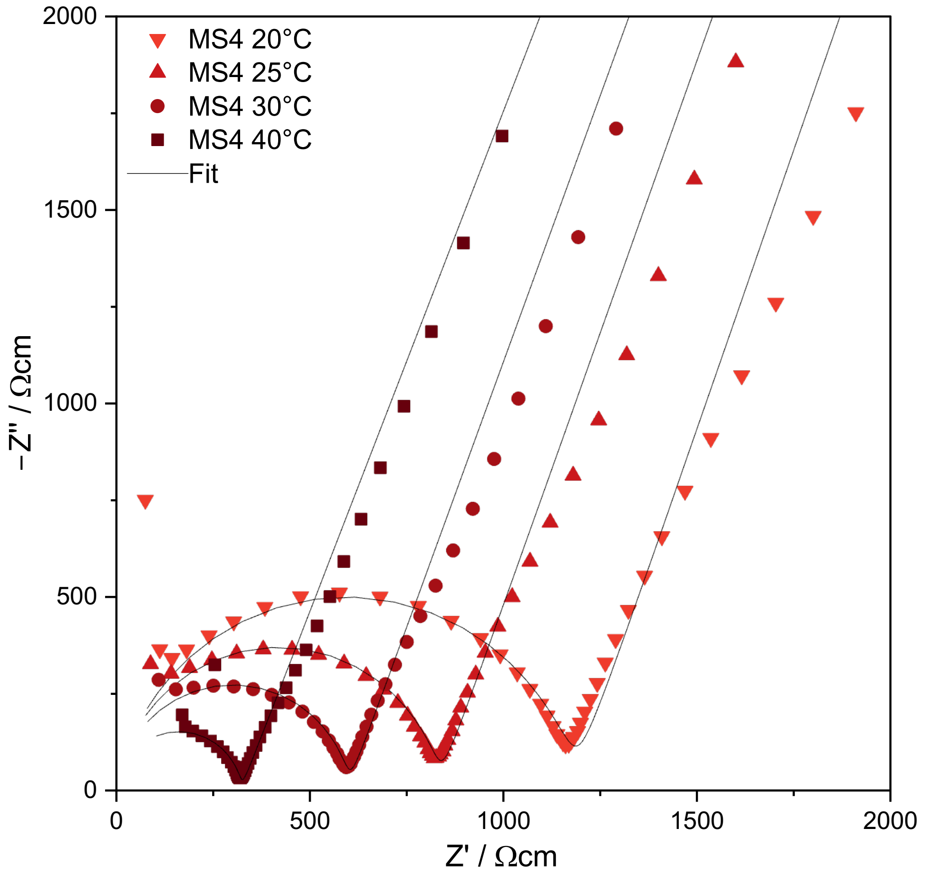

Figure 21. AC impedance spectrum of MS4 from 40°C to 20°C.

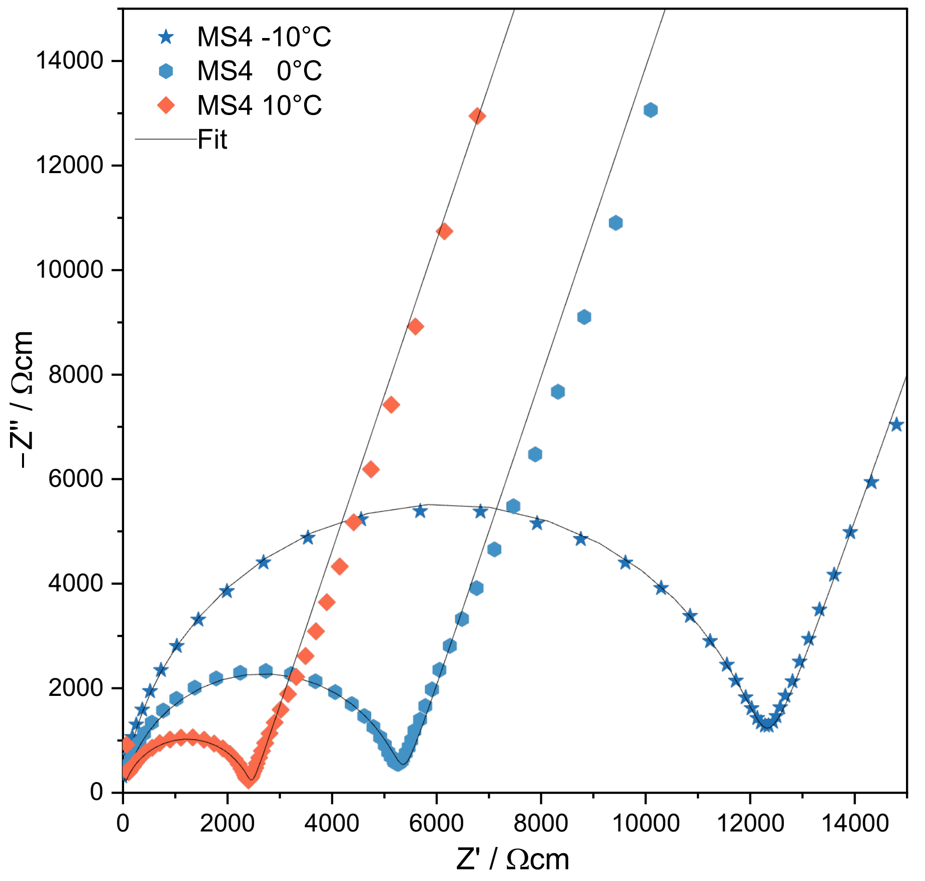

Figure 22. AC impedance spectrum of MS4 from 10°C to -10°C.

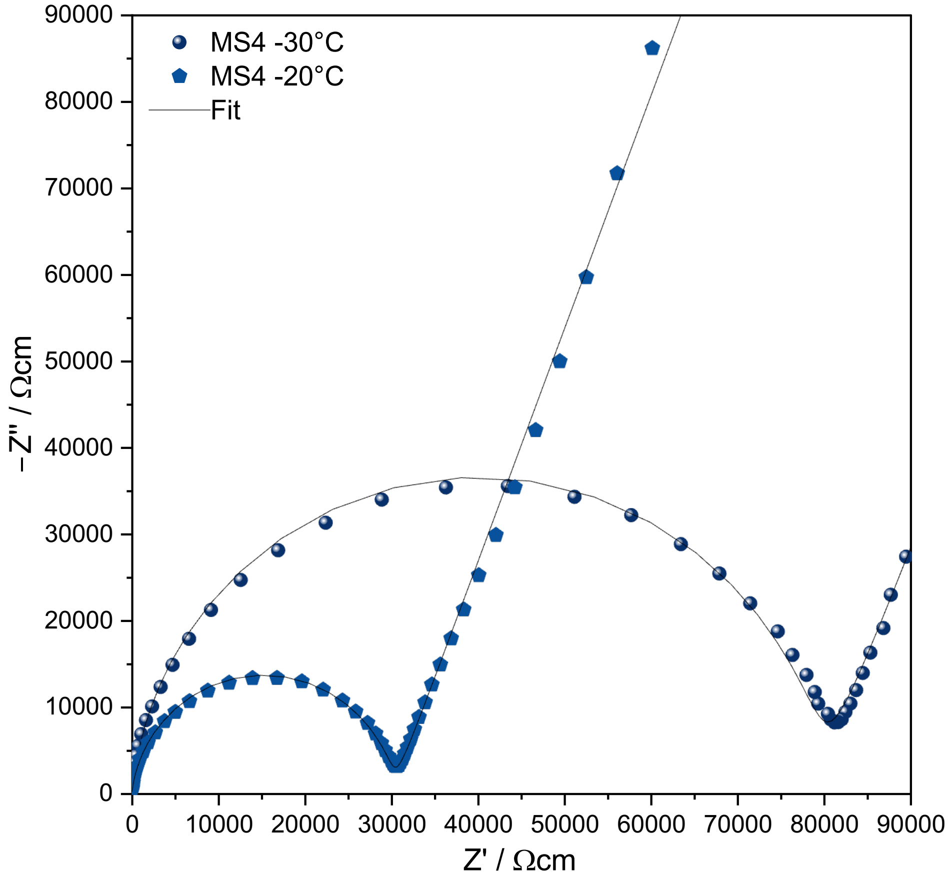

Figure 23. AC impedance spectrum of MS4 from -20°C to -30°C.

Table 1. Electrochemical Data for the measured samples at 25 °C.

	R1 / Ω	R2 / Ω	Q1 / C	Q2 / C	C1 / F	C2 / F	F1 / kHz	F2 / kHz	
DS1	1606.036								
DS2	4708.863	1427.732	5.419E-11	6.759E-8	2.455E-11	6.024E-9	1400	16.90	
DS3	2594.635								
DS4	1747.475								
MS1	3944.136								
MS2	1561.502	169.709	9.587E-11	3.902E-8	4.463E-11	3.902E-8	2200	32.00	
MS3	1412.088	319.582	2.615E-11	9.412E-8	2.615E-11	2.123E-8	4400	28.50	
MS4	4230.659								

	α_1	τ_1 / s	α_2	τ_2 / s	Q_{total} / C	C_{total} / F	α_{total}	τ_{total} / s	f_{total} / kHz
DS1					8.297E-11	3.121E-11	0.9412	3.610E-7	2770
DS2	0.9504	7.143E-7	0.7927	5.917E-5					
DS3					5.023E-11	3.162E-11	0.9728	2.841E-7	3520
DS4					7.514E-11	2.795E-11	0.9412	2.865E-7	3490
MS1					6.112E-11	2.700E-11	0.9491	7.246E-7	1380
MS2	0.9536	4.545E-7	1	3.125E-5					
MS3	1	2.273E-7	0.8749	3.509E-5					
MS4					1.120E-10	2.225E-11	0.9001	6.369E-7	1570
